# IFI16 and cGAS cooperate in the activation of STING during DNA sensing in human keratinocytes

**DOI:** 10.1038/ncomms14392

**Published:** 2017-02-13

**Authors:** Jessica F. Almine, Craig A. J. O'Hare, Gillian Dunphy, Ismar R. Haga, Rangeetha J. Naik, Abdelmadjid Atrih, Dympna J. Connolly, Jordan Taylor, Ian R. Kelsall, Andrew G. Bowie, Philippa M. Beard, Leonie Unterholzner

**Affiliations:** 1 Division of Cell Signalling and Immunology, School of Life Sciences, University of Dundee, Dundee DD1 5EH, UK; 2 Division of Biomedical and Life Sciences, Faculty of Health and Medicine, Lancaster University, Lancaster LA1 4YQ, UK; 3 The Pirbright Institute, Pirbright, Surrey GU24 0NF, UK; 4 Fingerprints Proteomics Facility, School of Life Sciences, University of Dundee, Dundee DD1 5EH, UK; 5 School of Biochemistry and Immunology, Trinity Biomedical Sciences Institute, Trinity College Dublin, Dublin 2, Ireland; 6 The Roslin Institute and The Royal (Dick) School of Veterinary Studies, University of Edinburgh, Edinburgh EH25 9RG, UK

## Abstract

Many human cells can sense the presence of exogenous DNA during infection though the cytosolic DNA receptor cyclic GMP-AMP synthase (cGAS), which produces the second messenger cyclic GMP-AMP (cGAMP). Other putative DNA receptors have been described, but whether their functions are redundant, tissue-specific or integrated in the cGAS-cGAMP pathway is unclear. Here we show that interferon-γ inducible protein 16 (IFI16) cooperates with cGAS during DNA sensing in human keratinocytes, as both cGAS and IFI16 are required for the full activation of an innate immune response to exogenous DNA and DNA viruses. IFI16 is also required for the cGAMP-induced activation of STING, and interacts with STING to promote STING phosphorylation and translocation. We propose that the two DNA sensors IFI16 and cGAS cooperate to prevent the spurious activation of the type I interferon response.

## Abstract (web summary)

The role of IFI16 as a DNA sensor is highly controversial. With support from a *Nature Communications* back-to-back publication from Jønsson *et al*., the authors here provide functional evidence that IFI16 is involved in DNA sensing via the cGAS-STING pathway in human keratinocytes.

Keratinocytes constitute the outermost layer of the skin, and as such are the first point of contact for many pathogens, including DNA viruses. Keratinocytes not only provide a physical barrier to infection and environmental insults but are also thought to function as sentinels of infection and injury that initiate and shape local immune responses^1^. However, their anti-viral defence mechanisms are relatively under-studied. Like many other cell types, keratinocytes are able to sense the presence of pathogens through pattern recognition receptors that detect pathogen-associated molecular patterns (PAMPs) as part of the immediate innate immune response to infection. Pattern recognition receptors include the Toll-like receptors at the cell surface and in endosomes, as well as intracellular receptors that sense the presence of viruses and intracellular bacteria inside infected host cells. The PAMPs that constitute the major tell-tale signs of viral infection are viral nucleic acids. Double-stranded RNA and single-stranded RNA with a 5′-triphosphate group for instance are detected as ‘foreign' by the cytosolic RNA receptors MDA5 and RIG-I, whereas pathogen-derived dsDNA can be detected by intracellular DNA receptors^2^.

Several cytosolic and nuclear DNA receptors promote the transcription of type I interferons, cytokines and chemokines upon recognition of DNA viruses, retroviruses and intracellular bacteria. An important DNA receptor in the cytosol is cyclic GMP-AMP synthase (cGAS), which catalyses the formation of the second messenger cyclic GMP-AMP (2′3′cGAMP, referred to as cGAMP throughout this manuscript)^3^,^4^. cGAMP then binds to the adaptor protein STING in the endoplasmic reticulum (ER), causing a conformational change in the STING dimer^5^. Activation of STING results in its relocalization from the ER to ER-Golgi intermediate compartments (ERGIC)^6^, where STING associates with TANK binding kinase 1 (TBK1). This interaction leads to the subsequent phosphorylation of STING by TBK1, which causes the recruitment of interferon regulatory factor 3 (IRF3)^7^, IRF3 phosphorylation and nuclear translocation. Together with nuclear factor κB (NF-κB), IRF3 is an important transcription factor for the activation of the *IFN-β* promoter, as well as for the expression of other cytokines, chemokines and IFN-stimulated genes during the innate immune response to viral infection.

Studies using cGAS-deficient mice, as well as mouse and human cell lines lacking cGAS expression, have provided evidence for a central role of cGAS during DNA sensing in a variety of infection contexts and cell types^8^. The discovery of cGAS has called into question the function of other, previously identified DNA receptors, which have also been described to detect viral dsDNA and activate STING^9^. One of the best described DNA sensors is interferon-γ-inducible protein 16 (IFI16), which shuttles between the nucleus and the cytosol, but is predominantly nuclear at steady state^10^,^11^. IFI16 is related to the inflammasome-inducing cytosolic DNA sensor AIM2 (ref. 12), and possesses an N-terminal pyrin domain and two HIN domains, which bind DNA in a sequence-independent manner^13^. IFI16 involvement in the type I interferon response to foreign DNA has been demonstrated using RNA interference (RNAi) approaches in a variety of mouse and human cells, and IFI16 and its mouse orthologue p204 have been shown to function in the innate immune response to DNA viruses such as HSV-1 in human and mouse myeloid cells, epithelial cells and fibroblasts^10^,^14^,^15^,^16^,^17^. IFI16 is also required for the response to infection with retroviruses such as HIV-1 in macrophages^18^ as well as to infection with intracellular bacteria such as *Listeria monocytogenes* in human myeloid cells^19^, and *Francisella novicida* in mouse macrophages^20^. In many of these cases, an essential role for cGAS has also been observed in the same cell type, during infection with the same pathogen or following stimulation with identical DNA ligands^15^,^18^,^19^,^20^,^21^. However, due to the reliance on RNAi approaches to diminish, rather than abolish IFI16 expression, the extent of redundancy or cooperation between IFI16 and cGAS has been difficult to ascertain. Furthermore, it has been reported that the entire family of murine AIM2-like receptors is dispensable for the interferon response to exogenous DNA in mice^22^, thus casting doubts over the role of IFI16 in the anti-viral response.

Here, we examine the role of IFI16 and cGAS in human keratinocytes, which are the target cells and first point of contact for a variety of DNA viruses. We use gene targeting to generate human immortalized HaCaT keratinocytes lacking IFI16 or cGAS, in order to investigate the function of these DNA receptors during the detection of exogenous DNA. We find that IFI16 and cGAS are not redundant during DNA sensing, but that both are required for the full activation of the innate immune response to exogenous DNA. Although the presence of cGAS is central for DNA sensing in keratinocytes, as it is in other cell types, IFI16 is closely integrated into the cGAS-cGAMP-STING signalling pathway by promoting the activation of STING in synergy with cGAMP. Thus, we propose that cGAS does not act in isolation, but rather cooperates with other factors such as IFI16 to activate STING in human cells.

## Results

### IFI16 is required for DNA sensing in HaCaT keratinocytes

**Figure 1:**
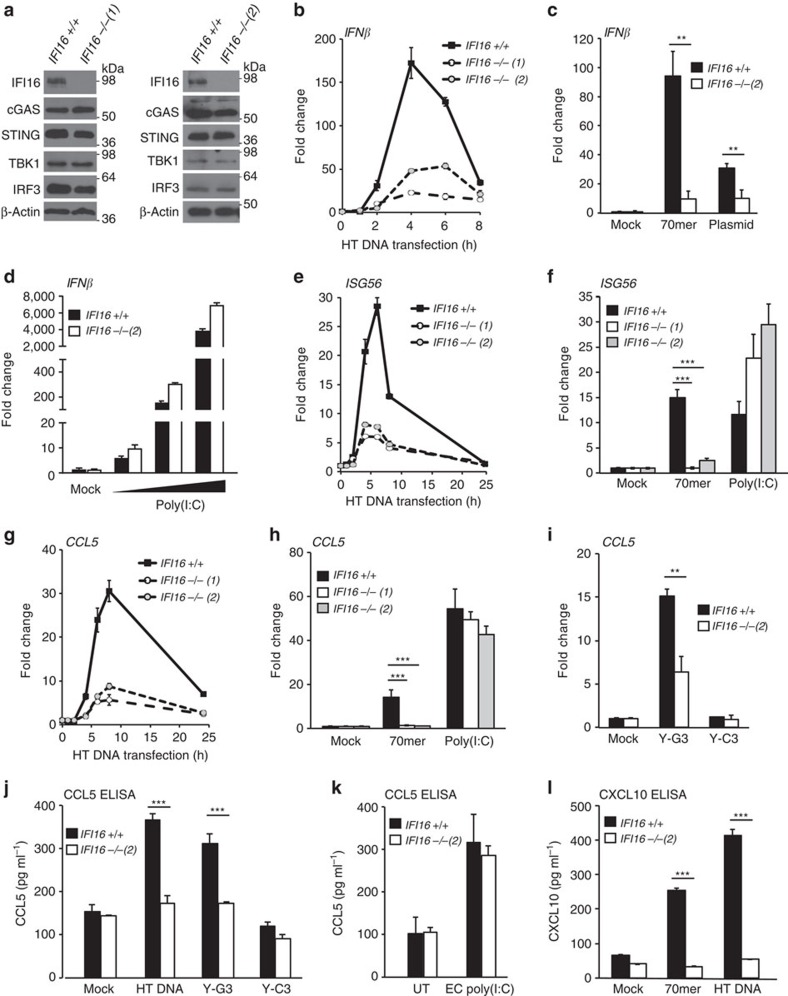
IFI16 is required for DNA but not RNA sensing in HaCaT keratinocytes. (**a**) Immunoblot analysis of wild-type (*IFI16* +/+) HaCaT and two *IFI16* −/− HaCaT clones. (**b**–**i**) Quantitative real-time PCR (qRT-PCR) analysis of mRNA expression levels normalized to *β-actin* mRNA and mock transfection in *IFI16* +/+ and *IFI16* −/− HaCaT cells, as indicated. (**b**) qRT-PCR analysis of *IFN-β* mRNA expression in IFI16+/+ and two IFI16 −/− HaCaT cells clones transfected with 1 μg ml^−1^ HT DNA for the times indicated. (**c**) qRT-PCR analysis of *IFN-β* mRNA 6 h post transfection with 1 μg ml^−1^ of a 70nt dsDNA oliogonucleotide (70mer) or circular pcDNA3.1 plasmid. (**d**) *IFN-β* mRNA induction 6 h after transfection with 1, 10 or 100 ng ml^−1^ poly(I:C). (**e**) Time course of *ISG56* mRNA expression following transfection with 1 μg ml^−1^ HT DNA. (**f**) *ISG56* mRNA expression 6 h post transfection with 1 μg ml^−1^ 70mer oligonucleotide or 100 ng ml^−1^ poly(I:C). (**g**) qRT-PCR analysis of *CCL5* mRNA expression following transfection with 1 μg ml^−1^ HT DNA for the times indicated. (**h**) Relative *CCL5* mRNA expression levels 6 h post transfection with 1 μg ml^−1^ 70mer oligonucleotide or 100 ng ml^−1^ poly(I:C). (**i**) *CCL5* mRNA expression levels 6 h post transfection with 1 μg ml^−1^ of Y-G3 or Y-C3 oligonucleotides. (**j**) Secreted CCL5 (Rantes) protein detected by ELISA in the supernatants of *IFI16* +/+ or *IFI16* −/− HaCaT cells transfected with 1 μg ml^−1^ HT DNA, Y-G3 or Y-C3 DNA for 24 h. (**k**) ELISA quantitation of CCL5 protein in supernatants from *IFI16* +/+ and *IFI16* −/− HaCaT cells stimulated with 5 μg ml^−1^ extracellular (EC) poly(I:C) added to the medium for 24 h. (**l**) ELISA quantitation of CXCL10 (IP-10) protein in supernatants of *IFI16* +/+ or *IFI16* −/− HaCaT cells transfected with 1 μg ml^−1^ 70mer oligonucleotide or HT DNA. All qRT-PCR and ELISA data are presented as mean values of biological triplicates. Error bars indicate s.d. *P<0.05, **P<0.01, ***P<0.001 Student's *t*-test. Data are representative of at least two experiments in two independent IFI16-deficient cell clones.

We used immortalized HaCaT keratinocytes as a model system to study the detection of viral DNA in a human cell type that is the initial point of contact for DNA viruses such as herpesviruses and poxviruses. Using transcription activator-like effector nuclease (TALEN) technology, two independent clonal cell lines were generated, where all IFI16 alleles contained insertions or deletions resulting in frameshift mutations. This resulted in the absence of detectable IFI16 protein expression as confirmed by Western blotting (Fig. 1a). HaCaT keratinocytes expressed cGAS, STING, TBK1 and IRF3 to similar extents in the presence and absence of IFI16 (Fig. 1a).

In order to assess the ability of HaCaT cells to respond to exogenous DNA, we transfected wild-type (*IFI16* +/+) HaCaT cells and the two *IFI16* −/− clones with herring testis (HT) DNA, and quantified the expression of *IFN-β* mRNA over time by real-time PCR. *IFI16* +/+ HaCaT keratinocytes generated a robust IFN-β response peaking at 4–6 h post DNA transfection. This response was severely blunted in both *IFI16* −/− clones (Fig. 1b). It has previously been suggested that IFI16 is dispensable for the early response to foreign DNA, but plays a role at later time points after DNA transfection in some cell types^15^,^23^. This does not seem to be the case in human keratinocytes, as the absence of IFI16 affected *IFN-β* mRNA expression as soon as induction was observed, at 2 h post stimulation (Fig. 1b). While we do observe a residual response in IFI16-deficient cells, this response occurs with similar kinetics as that in wild-type HaCaT cells. A similar deficiency in *IFN-β* mRNA production was observed following transfection with a 70 nt long dsDNA oligonucleotide (70mer, see ref. 10) or a circular dsDNA plasmid (Fig. 1c), or when HT DNA was introduced into cells by digitonin-mediated permeabilization (Supplementary Fig. 1a).

IFN-β expression induced by transfection of the dsRNA mimic poly(I:C) was not impaired in the absence of IFI16, even at the lowest poly(I:C) concentrations tested, and indeed often caused an enhanced response in IFI16-deficient cells (Fig. 1d). Both IFI16-deficient cell clones exhibited a similar impairment in the response to DNA, but not to poly(I:C) (Supplementary Fig. 1b,c), or to *in vitro* transcribed RNA containing 5′-triphosphates (Supplementary Fig. 1d). This demonstrates that HaCaT cells lacking IFI16 are still capable of mounting a type I interferon response, but are specifically impaired in their response to foreign DNA.

The activation of the *IFN-β* promoter relies on the transcription factors IRF3 and NF-κB, which are both activated by the adaptor protein STING. We found that the IRF3-dependent expression of the *interferon stimulated gene 56* (*ISG56*) was strongly impaired by the absence of IFI16 in response to DNA, but not poly(I:C) transfection (Fig. 1e,f). The same was true for the NF-κB-dependent transcription of *IL-6* mRNA (Supplementary Fig. 1e–g). IFI16 was also required for the DNA-, but not RNA-induced expression of the chemokines *CCL5* (Rantes, Fig. 1g,h) and *CXCL10* (IP-10, Supplementary Fig. 1h,i). IFI16-dependent *CCL5* mRNA induction was also observed following transfection of a short dsDNA oligonucleotide with single-stranded guanosine-containing overhangs (Y-G3 DNA), which has previously been implicated in the sequence-specific activation of cGAS in THP-1 monocytes^24^. In agreement with the mRNA expression data, we find that cells lacking IFI16 are unable to secrete CCL5 (Rantes) protein in response to transfected HT DNA or Y-G3 DNA (Fig. 1j), but CCL5 secretion is unaffected following stimulation with extracellular poly(I:C; Fig. 1k). Cells lacking IFI16 are also unable to induce CXCL10 (IP-10) secretion in response to exogenous DNA (Fig. 1l). Overall, we show that the innate immune response to exogenous DNA is strongly impaired in HaCaT cells lacking IFI16, while the response to poly(I:C) or to 5′-triphosphate-containing RNA is generally unaffected, or even enhanced. This confirms a specific involvement of IFI16 in the sensing of intracellular DNA.

### IFI16 is required for the response to DNA viruses

**Figure 2:**
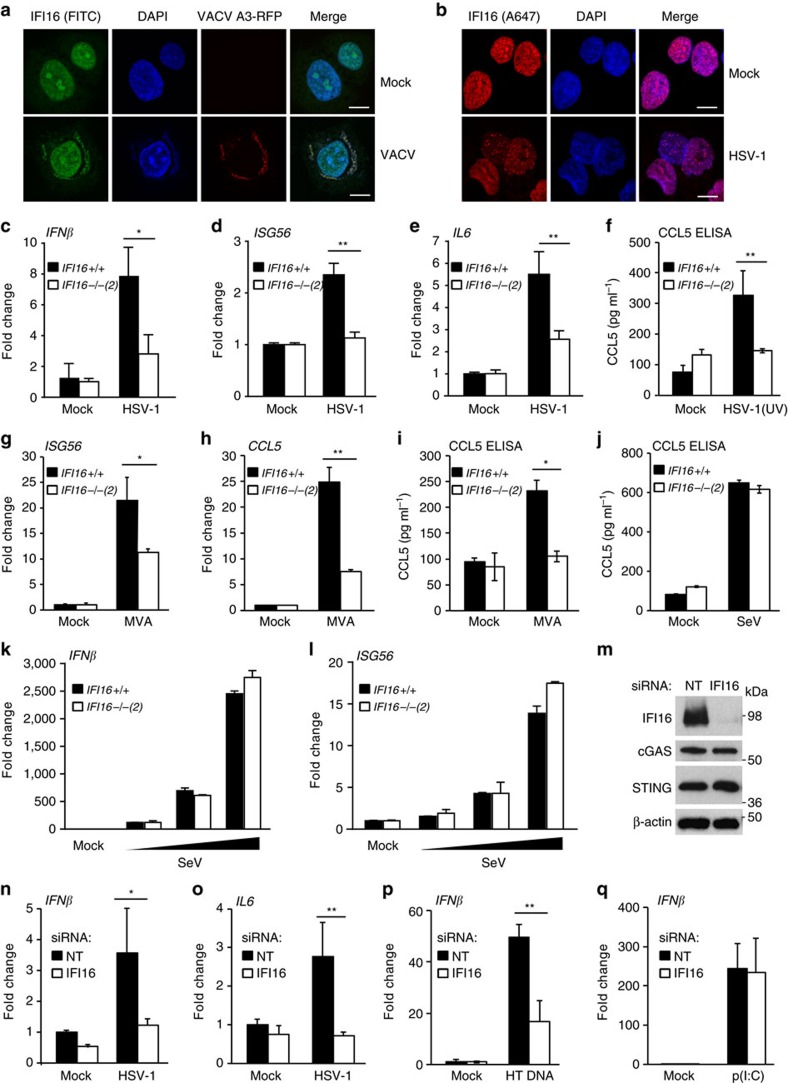
IFI16 is required for the innate immune response to DNA viruses. (**a**) Confocal imaging of HaCaT cells infected with VACV-A3-RFP (MOI=0.1) for 24 h and stained with FITC-labelled IFI16 antibody (green). A3-RFP is shown in red, DNA is stained with DAPI (blue). (**b**) Confocal imaging of HaCaT cells infected with HSV-1 (MOI=1) for 6 h and stained with anti-IFI16 antibody (red). DNA is visualized with DAPI (blue). Scale bars, 20 μm. (**c**–**e**) qRT-PCR analysis of IFI16 +/+ and IFI16 −/− HaCaT cells infected with HSV-1 (MOI=1) for 6 h. mRNA expression levels normalized to *β-actin* mRNA were determined for *IFNb* (**c**), *ISG56* (**d**) and *IL6* (**e**). (**f**) Secreted CCL5 protein from HaCaT cells infected with UV inactivated HSV-1 (MOI=5) for 24 h, quantified by ELISA. (**g**,**h**) qRT-PCR analysis of *ISG56* (**g**) and *CCL5* (**h**) mRNA expression in HaCaT cells infected with MVA (MOI=5) for 6 h. (**i**) ELISA quantitation of CCL5 protein in supernatants from HaCaT cells infected with MVA (MOI=5) for 24 h. (**j**) ELISA analysis of CCL5 protein from HaCaT cells infected with a Sendai virus (SeV) preparation containing defective viral particles (1:2,000 dilution) for 24 h. (**k**,**l**) qRT-PCR analysis of *IFNβ* (**k**) and *ISG56* (**l**) mRNA expression in HaCaT cells infected with Sendai virus (SeV) at dilutions of 1: 20 000, 1: 2,000 and 1:200 for 6 h. (**m**) Primary human keratinocytes (NHEK) were transfected with a non-targeting (NT) or IFI16-targeting siRNA pool for 48 h. Protein expression was examined by Western blotting. (**n**,**o**) NHEK were treated with siRNA pools for 48 h, and infected with HSV-1 (MOI=1) for 6 h. *IFN-β* (**n**) and *IL-6* (**o**) mRNA expression levels were quantified by qRT-PCR. (**p**,**q**) qRT-PCR analysis of IFN-β mRNA expression in MRC-5 human embryonic lung fibroblasts treated with siRNA pools for 48 h, and transfected for 6 h with 1 μg ml^−1^ HT DNA (**p**) or 100 ng ml^−1^ poly(I:C) (**q**). Data are representative of at least two independent experiments, and presented as mean values of biological triplicates, with error bars indicating s.d. *P<0.05, **P<0.01, ***P<0.001 Student's *t*-test.

Keratinocytes are natural host cells for many viruses including poxviruses such as vaccinia virus (VACV) and Modified Vaccinia virus Ankara (MVA) which replicate in the cytosol, and herpesviruses, such as herpes simplex virus 1 (HSV-1) which replicates in the nucleus of permissive cells. While IFI16 can shuttle between the nucleus and the cytosol^11^, it is predominantly nuclear at steady state in HaCaT keratinocytes (Fig. 2a), with low but detectable levels in the cytosol, as has been observed in other cell types^10^,^11^. We observed that during infection with VACV, endogenous IFI16 relocalized to viral factories in the cytosol, which also contain DNA and the VACV virus protein A3, as visualized during infection with VACV expressing an A3-mCherry fusion protein (Fig. 2a). During infection with HSV-1, which replicates in the nucleus, we observed a relocalization of IFI16 to nuclear puncta (Fig. 2b), which have previously been shown to be sites of HSV-1 replication^25^,^26^. Thus, during infection with DNA viruses IFI16 localizes to viral factories in both the nucleus and the cytosol, consistent with a role in the detection of foreign DNA in both compartments.

We next tested whether IFI16 is required for the sensing of DNA viruses. HSV-1 infection induced the expression of *IFN-β*, *ISG56* and *IL-6* mRNA in HaCaT keratinocytes, even though gene induction levels were modest, presumably due to the many countermeasures employed by wild-type HSV-1 to dampen the anti-viral response, which include the degradation of IFI16 and STING^25^,^27^,^28^. Nevertheless, the HSV-1-induced expression of *IFN-β*, I*SG56* and *IL-6* mRNA was impaired in IFI16-deficient cells (Fig. 2c–e and Supplementary Fig. 2a,b). HaCaT cells lacking IFI16 were also impaired in the secretion of CCL5 protein following infection with ultraviolet light-inactivated HSV-1 (Fig. 2f).

We were unable to detect an innate immune response to infection with VACV in HaCaT keratinocytes, as VACV also possess a large repertoire of inhibitors of innate immune signalling^29^. Thus, we examined the transcriptional response to the poxvirus Modified Vaccinia virus Ankara (MVA), an attenuated vaccine strain that lacks many of the immunomodulators of its relatives. MVA-induced *CCL5* and *ISG56* mRNA induction was significantly reduced in IFI16-deficient cells (Fig. 2g,h). Cells lacking IFI16 also secreted less CCL5 protein 24 h post infection with MVA (Fig. 2i).

We also infected HaCaT cells with a preparation of the Sendai virus (SeV) that contains a high proportion of defective viral particles allowing its RNA genome to be recognized by RIG-I^30^,^31^. SeV-induced CCL5 secretion was unaffected by the absence of IFI16 (Fig. 2j). Analogously, the induction of *IFN-β*, *ISG56* and *IL-6* mRNA expression in response to SeV was equally potent in wild-type and IFI16-deficient cells (Fig. 2k,l, Supplementary Fig. 2c,d).

We further confirmed the involvement of IFI16 in the sensing of DNA viruses in primary human cells by RNAi. Treatment of primary human keratinocytes from adult donors with a pool of four IFI16 siRNAs resulted in the potent knock-down of IFI16 protein expression (Fig. 2m). IFI16-depleted primary keratinocytes were unable to induce *IFN-β* or *IL-6* mRNA following infection with HSV-1 (Fig. 2n,o). Knock-down of IFI16 in embryonic lung fibroblast MRC-5 cells also reduced the interferon response to transfected DNA, but not to transfected poly(I:C) (Fig. 2p,q). This effect was also observed when individual IFI16-targeting siRNAs were used, confirming that the effects were not due to off-target effects of a particular siRNA sequence (Supplementary Fig. 2e).

### IFI16 is required for the DNA-induced activation of STING

We have previously shown that IFI16 can interact with the DNA sensing adaptor protein STING, and that p204, a mouse orthologue of IFI16, promotes the activation of IRF3 and NF-κB in mouse myeloid cells^10^,^14^. However, one study proposed that IFI16 can induce the transcription of *IFN-α* and *IFN-β* at the promoter level, and promotes IFN expression irrespective of stimulus^32^.

To confirm a role for IFI16 at the level of STING and transcription factor activation, we examined the individual steps in the signalling cascade activated by exogenous DNA. Upon stimulation with intracellular DNA, STING translocates away from the ER to the ERGIC and clusters in membrane-bound peri-nuclear foci^6^,^33^,^34^,^35^. STING signalling at the ERGIC results in the recruitment and activation of the kinase TBK1 (ref. 6). TBK1-mediated phosphorylation of STING is then thought to lead to the recruitment and activation of IRF3 (ref. 7), resulting in IRF3 phosphorylation, dimerization and nuclear translocation.

**Figure 3:**
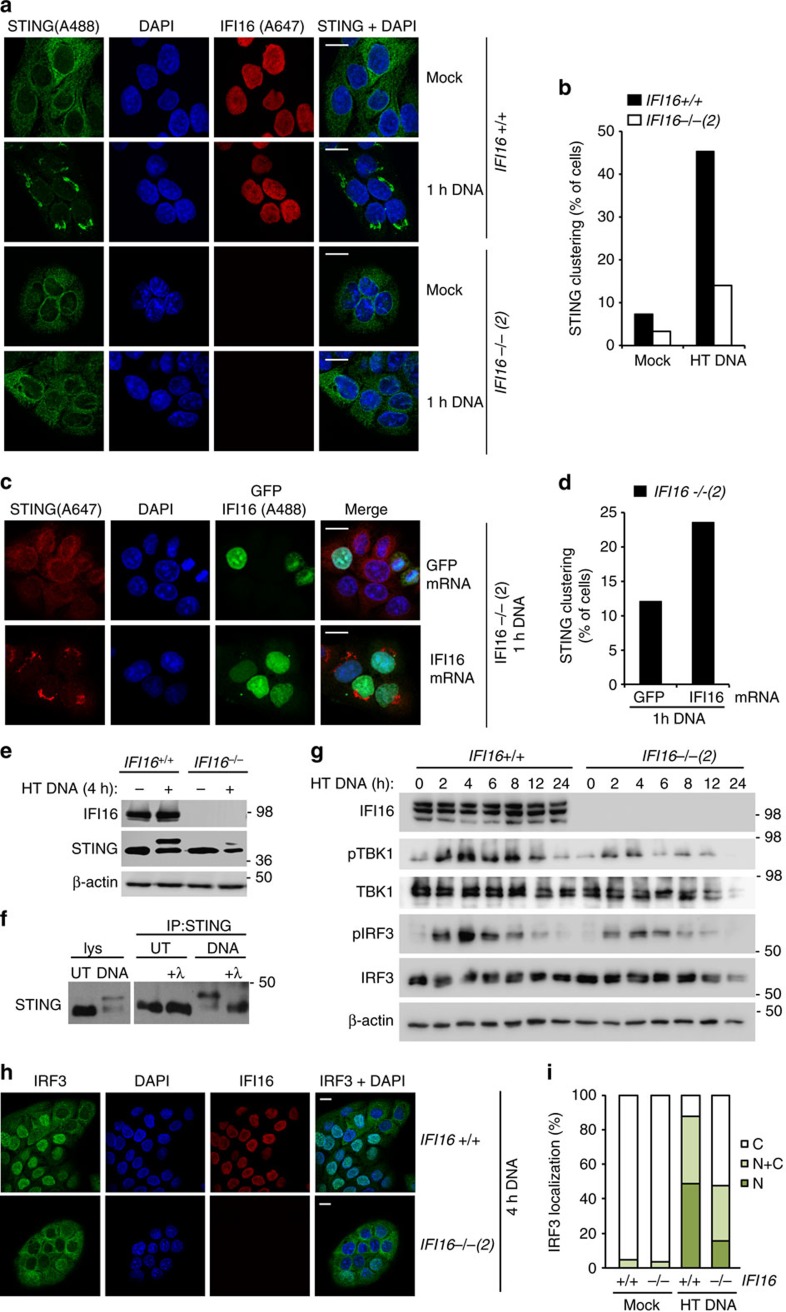
IFI16 is required for the DNA-induced activation of STING and IRF3. (**a**) Confocal analysis of *IFI16* +/+ and *IFI16* −/− HaCaT cells that were mock transfected or transfected for 1 h with 5 μg ml^−1^ HT DNA. Cells were stained for endogenous IFI16 (red) and STING (green). DNA is visualized with DAPI (blue). (**b**) Cells as in (**a**) were observed by confocal microscopy and scored for STING clustering. At least 200 cells were counted per sample. (**c**) Confocal analysis of *IFI16* −/− HaCaT cells reconstituted for 6 h with 1 μg ml^−1^ *in vitro* transcribed, capped and polyadenylated mRNA encoding GFP or IFI16, followed by transfection with 5 μg ml^−1^ HT DNA for 1 h. Cells were stained for STING (red), and DNA (DAPI, blue). GFP or AlexaFluor488-stained IFI16 are shown in green. (**d**) Cells as in **c** were scored for STING clustering, with at least 300 cells counted per sample. (**e**) Immunoblot analysis of HaCaT cells treated with 1 μg ml^−1^ HT DNA for 4 h, and probed for IFI16, STING and β-actin protein levels by Western blotting. (**f**) HaCaT cells were stimulated with 1 μg ml^−1^ HT DNA for 6 h or left untreated (UT). STING immunoprecipitates (IP) were treated with λ phosphatase where indicated, and analysed by western blotting. (**g**) Western blot analysis of IRF3 phosphorylation at Ser396 (pIRF3) and TBK1 phosphorylation at Ser172 (pTBK1) in HaCaT cells transfected with 1 μg ml^−1^ HT DNA for the times indicated. (**h**) HaCaT cells were transfected with 5 μg ml^−1^ HT DNA for 4 h, and the translocation of endogenous IRF3 was analysed by confocal microscopy. Cells were stained for IRF3 (green) and IFI16 (red), DNA is visualized with DAPI (blue). (**i**) Cells as in (**h**) were scored for predominately cytosolic (C), predominantly nuclear (N) and evenly distributed nuclear and cytosolic (N+C) localization of IRF3. At least 200 cells were counted per sample. Results are representative of at least two experiments each in two independent IFI16 −/− cell clones. Scale bars, 20 μm.

To place IFI16 in this signalling cascade, we first investigated the localization of endogenous STING protein in HaCaT keratinocytes by confocal microscopy. We found that STING relocalizes after 1 h stimulation with dsDNA, and moves from the ER to peri-nuclear foci in 46% of wild-type HaCaT cells (Fig. 3a,b). In HaCaT cells lacking IFI16, much fewer cells (12%) displayed DNA-induced STING clustering (Fig. 3a,b), suggesting that IFI16 affects the function of STING upon DNA transfection. This effect was also observed in a second *IFI16*-deficient cell clone (Supplementary Fig. 3a,b). Importantly, we were able to reconstitute *IFI16*-deficient cells with *in vitro* transcribed, capped and polyadenylated mRNA encoding *IFI16*. Reconstitution of cells with mRNA rather than expression plasmid allowed us to stimulate the cells by DNA transfection, and quantify STING translocation upon stimulation. *IFI16*-deficient cells transfected with mRNA expressing GFP displayed low levels of STING clustering after stimulation with exogenous DNA (12% of cells), like the *IFI16* −/− cells before mRNA transfection (Fig. 3c,d). In I*FI16*-deficient cells reconstituted with mRNA encoding *IFI16*, more cells (24%) showed DNA-induced STING translocation (Fig. 3c,d). This shows unequivocally that IFI16 is involved in the DNA-induced translocation of STING.

The presence of exogenous DNA induces the phosphorylation of STING by TBK1 and other kinases^7^,^36^. We observe the appearance of a more slowly migrating STING band by SDS–polyacrylamide gel electrophoresis (SDS–PAGE) following transfection with HT DNA and Y-G3 DNA in wild-type HaCaT cells, which is reduced in the absence of IFI16 (Fig. 3e and Supplementary Fig. 3c). This band is indeed a phosphorylated form of STING, as shown by STING immunoprecipitation followed by treatment with *λ* phosphatase (Fig. 3f). Thus, IFI16 plays a role in the DNA-induced phosphorylation of STING.

We also tracked the phosphorylation of TBK1 (at Serine 172) and IRF3 (at Serine 396) over time after DNA transfection. Phosphorylation of TBK1 and IRF3 peaked at 4 h post DNA transfection in wild-type HaCaT cells. TBK1 and IRF3 phosphorylation levels were much reduced, but not completely absent in cells lacking IFI16 (Fig. 3g), consistent with a reduced transcriptional response to exogenous DNA. In agreement with the unimpaired transcriptional response to poly(I:C), the phosphorylation of TBK1 and IRF3 induced by poly(I:C) was able to proceed in the absence of IFI16 (Supplementary Fig. 3d). Both IFI16-deficient cell clones also showed impaired translocation of IRF3 to the nucleus at 4 h post transfection, as observed by confocal microscopy (Fig. 3h,i and Supplementary Fig. 3e,f). Taken together, our data indicate that IFI16 acts ‘upstream' of STING and transcription factor activation during DNA sensing, consistent with a role as *bona fide* co-receptor in this signalling pathway.

### DNA sensing in HaCaT keratinocytes also requires cGAS

**Figure 4:**
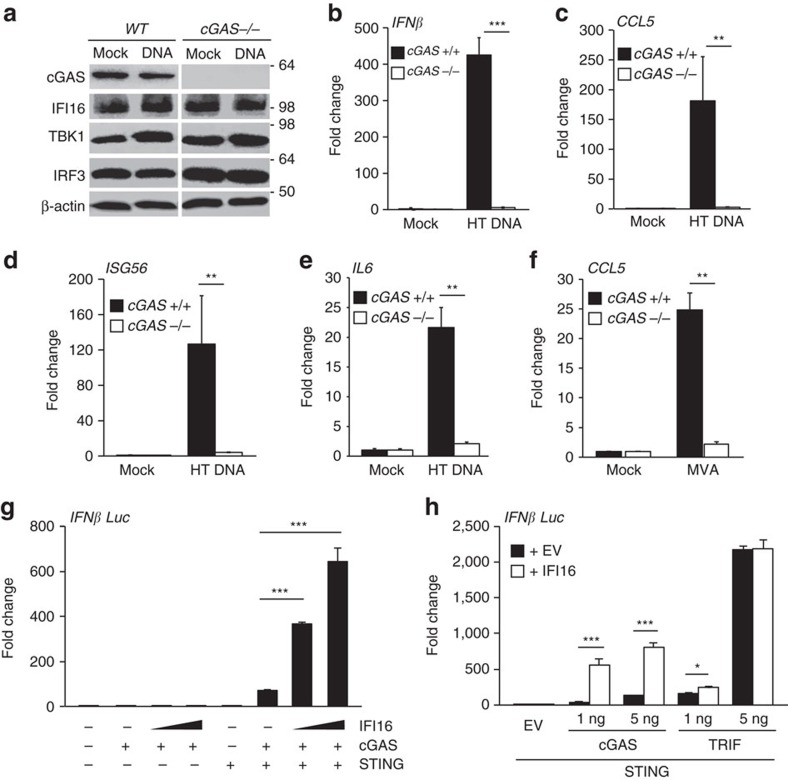
cGAS is required for the innate immune response to DNA in HaCaT keratinocytes. (**a**) Immunoblot analysis of wild-type (WT) and *cGAS* −/− HaCaT cells, mock transfected or transfected with 1 μg ml^−1^ HT DNA for 6 h. (**b**–**e**) qRT-PCR analysis of *cGAS* +/+ and *cGAS* −/− HaCaT cells that were mock transfected or transfected with 1 μg ml^−1^ HT DNA for 6 h. mRNA levels were normalized to *β-actin* mRNA levels and mock transfections. *IFNβ* (**b**), *CCL5* (**c**), *ISG56* (**d**) and *IL6* (**e**) mRNA levels are shown. (**f**) qRT-PCR analysis *CCL5* mRNA from *cGAS* +/+ and *cGAS* −/− HaCaT cells infected with MVA (MOI=5) for 6 h. (**g**) HEK293T cells were transfected with a firefly luciferase reporter construct under the control of the *IFN-β* promoter, a Renilla luciferase transfection control, 10 ng STING-Flag plasmid, 1 ng cGAS-Flag and 35 or 70 ng HA-IFI16 expression plasmids, as indicated. Firefly luciferase activity was measured 24 h post transfection, and normalized to Renilla luciferase activity. (**h**) HEK293T cells were transfected with a firefly luciferase reporter construct under the control of the *IFNβ* promoter, Renilla luciferase transfection control and 10 ng STING-Flag expression plasmid. In addition, 1 or 5 ng cGAS or TRIF expression constructs were co-expressed with 35 ng HA-IFI16 plasmid or empty vector, as indicated. Relative Firefly luciferase activity was quantified 24 h post transfection. Data are representative of at least three independent experiments, and presented as mean values of triplicate samples. Error bars indicate s.d. *P<0.05, **P<0.01, ***P<0.001 Student's *t*-test.

HaCaT keratinocytes also express cGAS (Fig. 1a). In order to assess whether the function of cGAS is as critical during DNA sensing in human keratinocytes, as it is in many other cell types^8^, we generated HaCaT cells lacking *cGAS*, using a CRISPR-Cas9 nickase approach. *cGAS*-deficient HaCaT cells still contained similar IFI16 protein levels as wild-type cells (Fig. 4a). Thus, deletion of cGAS in HaCaT cells does not automatically result in the reduction of IFI16 protein levels which has been observed in other cell contexts^15^,^20^, and the relative function of the two DNA sensors can be examined in isolation.

We find that cGAS-deficient HaCaT cells are unable to induce *IFN-β*, *CCL5*, *ISG56* and *IL-6* mRNA at 6 h post stimulation with transfected DNA (Fig. 4b–e). cGAS-deficient cells are also impaired in their response to infection with the cytosolic DNA virus MVA (Fig. 4f), as measured by *CCL5* mRNA induction. Thus, as in many other cells, cGAS is essential for the response to foreign DNA and DNA viruses in HaCaT keratinocytes. Given that we have shown here that IFI16 also has an important role in the same cells and in response to the same DNA ligands and viruses, our data suggest that IFI16 and cGAS each have important, but functionally different, roles in the innate immune response to DNA, and need to cooperate to achieve full activation of an anti-viral response.

To test whether the cooperation between IFI16 and cGAS can also be observed in HEK293T cells, which do not express endogenous STING and are unable to mount an innate immune response upon DNA transfection^10^,^37^, we transfected HEK293T cells with expression constructs encoding *STING*, *cGAS* and *IFI16* and measured *IFNβ* promoter activation using luciferase assays. We found that IFI16 synergizes with cGAS and STING in the activation of the *IFNβ* promoter in a dose-dependent manner (Fig. 4g). Furthermore, the activities of IFI16 and cGAS were critically dependent on the presence of STING in this system (Fig. 4g). IFI16 did not synergize to the same extent with other signalling factors such as the TLR3 adaptor protein TRIF, even when STING was co-expressed (Fig. 4h). This indicates that the strong synergy between IFI16 and cGAS is specific to their roles in the DNA sensing pathway, rather than simply being due to an additive effect of two independent IFN-inducing factors.

### IFI16 interacts with cGAS in a DNA-dependent manner

The molecular function of cGAS in the DNA sensing pathway is well-defined. Upon recognition of DNA, cGAS catalyses the production of the second messenger cGAMP from ATP and GTP. cGAMP then binds to STING dimers, resulting in a conformational change in STING that is thought to contribute to STING activation^5^. We find that IFI16 also influences STING phosphorylation and translocation in response to DNA (Fig. 3a–f). To place the function of IFI16 in the context of the cGAS-cGAMP-STING pathway, we examine whether IFI16 plays a role in the DNA-induced production of cGAMP, and/or in the cGAMP-induced activation of STING.

**Figure 5:**
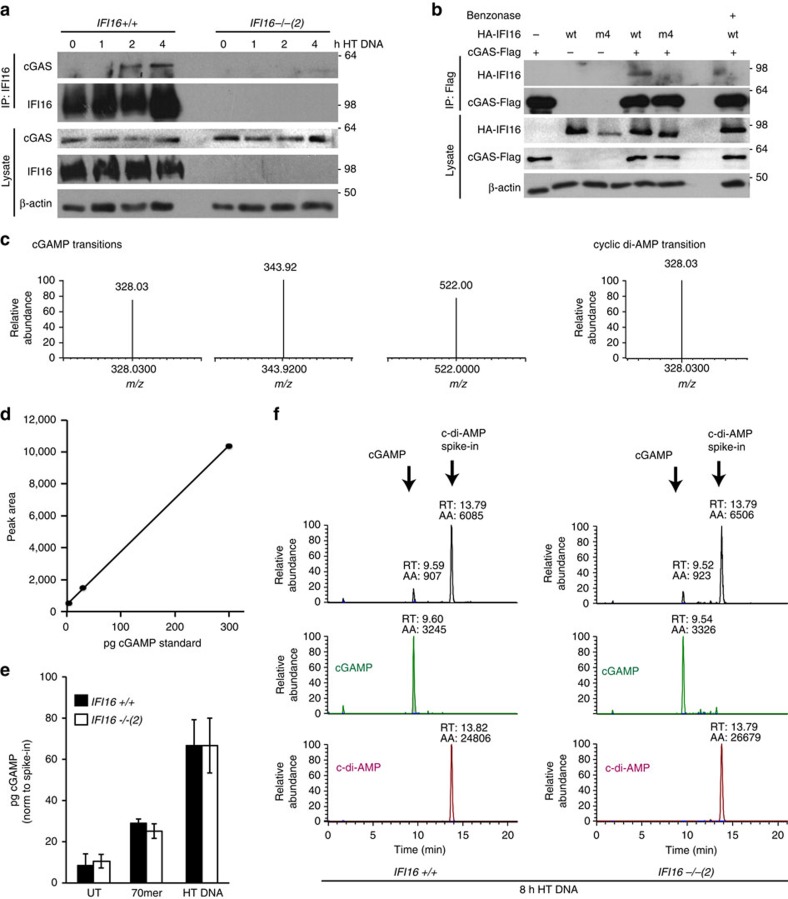
IFI16 interacts with cGAS but does not affect cGAMP production. (**a**) *IFI16* +/+ or *IFI16* −/− HaCaT cells were stimulated with 5 μg ml^−1^ HT DNA for the times indicated, and IFI16 was immunoprecipitated from cell lysates. Lysates and immunoprecipitates (IP) were analysed by SDS–PAGE and western blotting. (**b**) HEK293T cells were transfected with constructs for the expression of cGAS-FLAG and HA-IFI16, either wild-type (wt) or DNA-binding mutant (m4), as indicated. 24 h post transfection, cells were subjected to lysis and immunoprecipitation using FLAG antibody. Immunoprecipitates were washed, and treated with benzonase where indicated. Lysates and immunoprecipitates (IP) were analysed by SDS–PAGE and western blotting. (**c**) Multiple reaction monitoring transitions for cGAMP and cyclic di-AMP, used for the quantification of endogenous cGAMP and internal standard cyclic di-AMP. *m/z*, mass/charge ratio of fragment ions. (**d**) Standard curve for synthetic cGAMP spiked into cell lysates before sample preparation and liquid chromatography and mass spectrometry (LC-MS) analysis. (**e**) *IFI16* +/+ and *IFI16* −/− HaCaT cells were treated with 1 μg ml^−1^ 70mer oligonucleotide or HT DNA for 8 h, followed by lysis in methanol, spike-in of c-di-AMP and sample preparation. cGAMP levels were determined by LC-MS, and normalized to c-di-AMP levels to account for losses in sample preparation and injection. Data are representative of at least four experiments; values are shown as mean of triplicate samples, with error bars representing s.d. (**f**) Total and extracted ion chromatogram of cGAMP and cyclic di-AMP in representative samples from (**e**), showing *IFI16* +/+ and *IFI16* −/− cells treated with HT DNA for 8 h. AA, integral peak area; RT, retention time.

We first tested whether IFI16 and cGAS would form a complex during DNA sensing. We were able to detect an interaction between endogenous IFI16 and cGAS that was enhanced by stimulation with DNA (Fig. 5a). We could also detect the interaction in FlipIn HEK293 cells expressing GFP-IFI16, but not GFP alone (Supplementary Fig. 4a) and in HEK293T cells expressing HA-tagged IFI16 and Flag-tagged cGAS (Fig. 5b). The interaction between the two proteins is facilitated by DNA as a binding platform, as cGAS does not interact with a IFI16 protein containing several point mutations that impair its ability to bind DNA (IFI16-m4, described in ref. 13) (Fig. 5b). Furthermore, treatment of the IFI16-cGAS complex with benzonase, a nuclease which degrades DNA and RNA, also reduced the interaction (Fig. 5b). Thus, IFI16 and cGAS are brought together by assembling on exogenous DNA.

### IFI16 is not required for cGAMP production in HaCaT cells

We next tested whether IFI16 would be able to influence cGAS function in production of the second messenger cGAMP. To measure the production of cGAMP during DNA sensing, we quantified endogenous cGAMP levels in cell extracts after DNA stimulation using a liquid chromatography and mass spectrometry (LC-MS/MS) approach outlined in Supplementary Fig. 4b. Multiple reaction monitoring allowed us to unambiguously identify cGAMP, as well as cyclic-di-AMP which we used as internal spike-in control to account for losses during the sample preparation and injection. Three *m/z* transitions were used for the identification of cGAMP, and one for c-di-AMP (Fig. 5c), allowing us to accurately detect synthetic cGAMP and c-di-AMP standards (Supplementary Fig. 4c), and quantify cGAMP in a background of processed cell lysates with pg sensitivity (standard curve in Fig. 5d). Unstimulated HaCaT cells contain low, but detectable amounts of cGAMP (Fig. 5e and Supplementary Fig. 4d). Following stimulation with HT DNA or VACV 70mer oligonucleotide, cGAMP levels increase in both wild-type and IFI16-deficient HaCaT cells (Fig. 5e,f and Supplementary Fig. 4e). Treatment of cell extracts with snake venom phosphodiesterase removes the cGAMP peak following DNA stimulation (Supplementary Fig. 4f), as would be predicted^38^. Thus, we conclude that IFI16 is not required for cGAMP production in HaCaT keratinocytes.

### IFI16 is required for the response to exogenous cGAMP

We next tested whether IFI16 affects the activation of STING by cGAMP. Cells can be stimulated by the intracellular delivery of cGAMP, thus by-passing cGAS function and the production of endogenous cGAMP.

**Figure 6:**
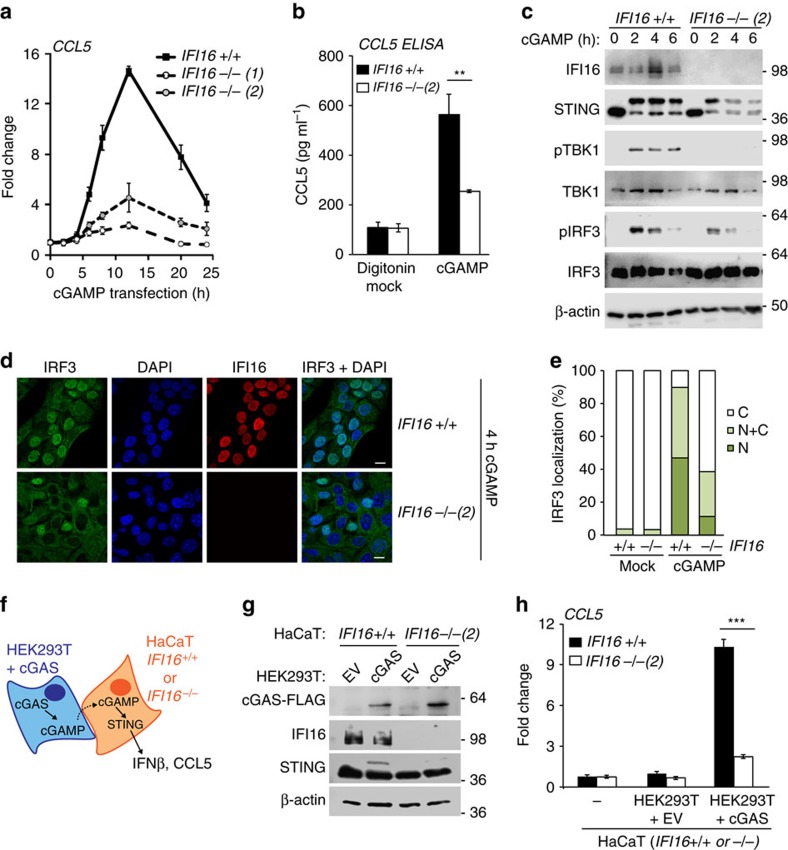
IFI16 is required for cGAMP-induced STING activation. (**a**) *IFI16* +/+ and *IFI16* −/− HaCaT cells were transfected with 20 μg ml^−1^ synthetic cGAMP, and *CCL5* mRNA induction was analysed by qRT-PCR at the time points indicated, and normalized to *β-actin* mRNA. (**b**) *IFI16* +/+ and *IFI16* −/− HaCaT cells were infused with 15 μM cGAMP by digitonin-mediated permeabilization, and CCL5 protein in supernatants was quantified by ELISA 24 h post stimulation. (**c**) HaCaT cells were permeabilized with digitonin and infused with 15 μM cGAMP for 2, 4 or 6 h. Phosphorylation of STING, of IRF3 at Ser396 (pIRF3) and TBK1 at Ser172 (pTBK1) was analysed by SDS–PAGE and western blotting. (**d**) HaCaT cells were transfected with 20 μg ml^−1^ cGAMP for 4 h, and the translocation of endogenous IRF3 was observed by confocal microscopy. Cells were stained for IRF3 (green), IFI16 (red) and DNA (DAPI, blue). Scale bar, 20 μm. (**e**) Cells as in **d** were scored for predominantly cytosolic (C), predominantly nuclear (N) and evenly distributed nuclear and cytosolic (N+C) localization of IRF3. At least 200 cells were counted per sample. (**f**) Schematic representation of the co-culture of HaCaT cells with cGAS-expressing HEK293T cells. Endogenously produced cGAMP can diffuse through gap junctions from the cGAS-expressing producer HEK293T cells to HaCaT cells, where it can bind STING to induce an innate immune response. (**g**,**h**) HEK293T cells were transiently transfected with a cGAS-FLAG expression construct or empty vector (EV) for 6 h, then co-cultured with *IFI16* +/+ or *IFI16* −/− HaCaT cells for 18 h. (**g**) Immunoblot analysis of cGAS-FLAG, IFI16 and STING protein expression in the co-culture. (**h**) qRT-PCR analysis of *CCL5* mRNA expression in *IFI16* +/+ or *IFI16* −/− HaCaT cells grown in monoculture (−) or co-cultured with HEK293T cells expressing cGAS-FLAG or empty vector (EV). Data show means of triplicate samples with s.d. Shown are representatives of at least two independent experiments each in two IFI16 −/− cell clones.

In order to assess the function of IFI16 in this context, we transfected HaCaT cells with synthetic 2′3′ cGAMP, and quantified the gene expression response over time. The delivery of synthetic cGAMP induced the expression of *CCL5* and *ISG56* mRNA in wild-type HaCaT cells, peaking at 12 and 6 h post transfection. *IFI16*-deficient cells exhibited a severely blunted response that occurred with similar kinetics to the response in wild-type cells (Fig. 6a and Supplementary Fig. 5a). As lipofection has also been described to induce a STING-dependent innate immune response in some cells^39^, we tested other means of delivering cGAMP. A similar reduction in cGAMP-induced gene expression was observed when cGAMP was infused into the cells by digitonin-mediated permeabilization (Supplementary Fig. 5b,c). *IFI16*-deficient cells also secreted less CCL5 protein quantified by ELISA (Fig. 6b). In analogy to our observations in cells stimulated by DNA transfection, *IFI16* deficiency also impaired the phosphorylation of STING, TBK1 and IRF3 following stimulation with cGAMP (Fig. 6c), and the translocation of IRF3 to the nucleus (Fig. 6d,e).

Finally, we tested the response of HaCaT cells to endogenously produced cGAMP delivered though gap junctions. For this, we over-expressed cGAS in HEK293T cells, which acted as producer cells for endogenous cGAMP, and co-cultured these with wild-type or *IFI16*-deficient HaCaT cells (schematic representation in Fig. 6f). The expression levels of FLAG-tagged cGAS in the co-culture were confirmed by western blotting (Fig. 6g). As HEK293T cells do not express STING, they cannot respond to the cGAMP they produce and are not stimulated by the over-expression of cGAS alone (Fig. 4g). However, neighbouring HaCaT cells that are in direct contact with the cGAS-expressing HEK293T cells take up cGAMP through gap junctions, resulting in the activation of STING and the induction of an innate immune response in the HaCaT cells. Co-culture with cGAS-expressing HEK293T cells, but not HEK293T cells transfected with empty vector, induced the phosphorylation of endogenous STING in the HaCaT cells, which was reduced in HaCaT cells lacking IFI16 (Fig. 6g). As a consequence of STING activation, HaCaT cells co-cultured with cGAS-expressing HEK293T cells induce the expression of CCL5 mRNA, compared with HaCaT monocultures or co-cultures with HEK293T cells containing empty vector (Fig. 6h). In agreement with our data using synthetic cGAMP, CCL5 mRNA levels induced by endogenous cGAMP were significantly lower in *IFI16*-deficient HaCaT cells (Fig. 6h), despite similar levels of cGAS expression in the co-culture (Fig. 6g). IFI16 was also required for the expression of *ISG56* and *IFN-β* in these co-culture experiments (Supplementary Fig. 5d–f). Taken together, we find that IFI16 is required for the response to cGAMP, whether delivered into the cells by permeabilization, transfection or through gap junctions from neighbouring cells.

### IFI16 provides an additional signal for STING activation

**Figure 7:**
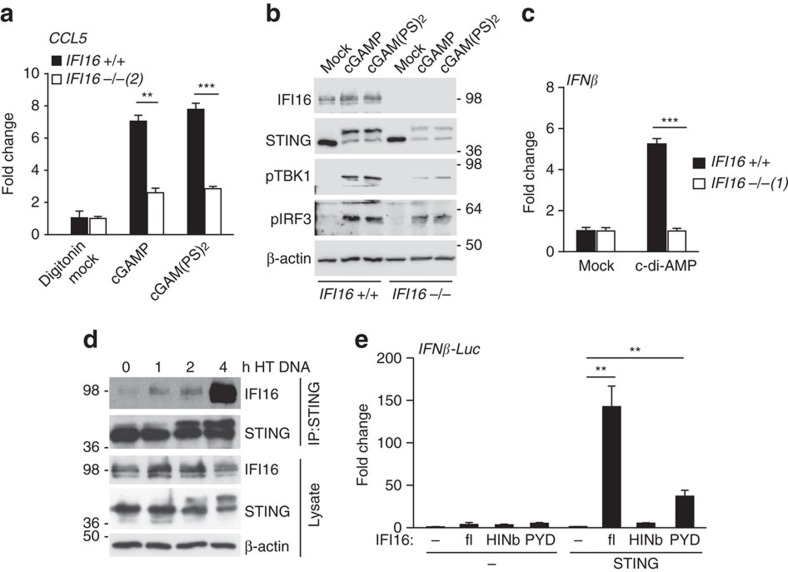
IFI16 acts on STING to promote its activation by cyclic di-nucleotides. (**a**) *IFI16* +/+ or *IFI16* −/− HaCaT cells were permeabilized with digitonin and infused with 15 μM cGAMP or its non-hydrolysable analogue cGAM(PS)_2_ for 6 h. *CCL5* mRNA expression was analysed by qRT-PCR. (**b**) Cells were permeabilized and infused with 15 μM cGAMP or cGAM(PS)_2_ for 4 h, and lysates were analysed by western blotting for phosphorylation of STING, TBK1 at Ser172 (pTBK1) and IRF3 at Ser396 (pIRF3). (**c**) Cells were transfected with 100 μg ml^−1^ cyclic di-AMP for 6 h, and *IFN-β* mRNA levels were quantified by qRT-PCR. (**d**) STING was immunoprecipitated from HaCaT cells transfected with 5 μg ml^−1^ HT DNA for the times indicated. Lysates and immunoprecipitates (IP) were analysed by SDS–PAGE and western blotting. (**e**) HEK293T cells were transfected with a firefly luciferase reporter construct under the control of the *IFNβ* promoter, a Renilla luciferase transfection control, 2 ng STING-FLAG plasmid and 150 ng empty vector (EV) or IFI16 expression constructs as indicated: full-length IFI16 (fl), the IFI16 HINb domain (HINb), or the IFI16 pyrin domain (PYD). Firefly luciferase activity was measured 24 h post transfection, and normalized to Renilla luciferase activity. Data are representative of at least two independent experiments. qRT-PCR and luciferase data are expressed as means of triplicate samples; error bars represent s.d. *P<0.05, **P<0.01, ***P<0.001 Student's *t*-test.

The observed effects of IFI16 on cGAMP-induced STING activation could potentially be explained by a role of IFI16 in the stabilization of cGAMP. For this reason, we tested whether the use of a non-hydrolysable analogue of cGAMP, cGAM(PS)_2_ (ref. 40), would overcome the effect of IFI16 on cGAMP-induced activation of an innate immune response. We found that *CCL5* mRNA expression following the exposure of cells to cGAMP or its non-hydrolysable analogue was equally affected by the absence of IFI16 (Fig. 7a). Analogously, STING phosphorylation and the activation of TBK1 and IRF3 were reduced in *IFI16*-deficient cells, regardless of whether the cells were stimulated with cGAMP or cGAM(PS)_2_ (Fig. 7b). While we cannot formally exclude a role of IFI16 in affecting cGAMP turnover, our results indicate that IFI16 has an important function in cGAMP-induced STING activation that is independent of cGAMP hydrolysis.

We also examined whether IFI16 is required for the response to other cyclic di-nucleotides that are sensed by STING. STING can detect molecules such as cyclic di-AMP and cyclic di-GMP which are produced by bacteria, and constitute a PAMP during infection with intracellular pathogens^37^. Some common STING sequence variants display impaired sensing of bacterial cyclic di-nucleotides^41^. Sequencing of STING cDNA in HaCaT cells did not reveal the presence of alleles containing such sequence polymorphisms, and, in agreement with this, HaCaT cells can respond to the transfection of synthetic cyclic di-AMP. The response to cyclic di-AMP was also dependent on IFI16 (Fig. 7c). Thus, the involvement of IFI16 in STING activation is not limited to the DNA sensing pathway, but also encompasses the innate immune response to bacterial cyclic di-nucleotide PAMPs in human keratinocytes.

We next tested the interaction between IFI16 and STING during DNA sensing. Using co-immunoprecipitation, we can detect a constitutive weak interaction between endogenous STING and IFI16 in HaCaT cells, and complex formation increases in the hours following DNA transfection (Fig. 7d). However, we do not observe a clear co-localization of IFI16 and STING in DNA-stimulated HaCaT cells (see Fig. 3a), suggesting that the association between the two proteins is likely dynamic.

Given that IFI16 binds to STING and synergizes with cGAMP in STING activation, we tested whether IFI16 would be able to influence STING function in the absence of cGAS and cGAMP. When IFI16 is transiently expressed in HEK293T cells in the presence of a luciferase reporter system driven by the *IFNβ* promoter, IFI16 is only able to activate the *IFNβ* promoter if STING is also co-expressed (Fig. 7e). IFI16 contains two C-terminal HIN domains which bind DNA^13^ and an N-terminal pyrin domain (PYD) which is thought to mediate its signalling functions. We found that over-expression of the PYD alone is able to drive STING activation in this assay, while expression of the DNA-binding HINb domain is not (Fig. 7e). We have previously shown that the DNA-binding function of IFI16 is required for full STING activation in the context of the full-length IFI16 protein in this assay, where plasmid DNA likely provides the stimulus^13^. This correlates with the DNA-induced interaction between endogenous IFI16 and STING that we observe under more physiological conditions in HaCaT keratinocytes (Fig. 7d). Over-expression of the pyrin domain likely drives the activation of STING constitutively, by-passing the requirement for DNA detection by the HIN domain. Taken together, we find that IFI16 acts on STING via its pyrin domain, and cooperates with cGAMP and other cyclic di-nucleotides to promote the phosphorylation and translocation of STING.

## Discussion

The function of IFI16 as a receptor for foreign DNA during infection with DNA viruses and intracellular bacteria is supported by a large body of evidence, mostly relying on the use of RNAi approaches^42^. It has been reported that p204, a mouse orthologue of IFI16, cooperates with cGAS during *Francisella novicida* infection in murine RAW264.7 monocytic cells^20^, and synergy between IFI16 and cGAS has also been observed during *Listeria monocytogenes* infection in human myeloid cells, and during HSV-1 infection in primary human foreskin fibroblasts^15^,^19^, using RNAi approaches to study the effect of IFI16 and cGAS depletion. However, one study suggested that IFI16 may have a more generic function in the transcriptional activation of type I IFN regardless of stimulus^32^, and it has recently been shown that the locus containing all murine homologues of IFI16 is dispensable for DNA sensing in mice^22^. This study also reported that pools of gene targeted human fibroblasts with low or undetectable levels of IFI16 protein displayed unimpaired *IFNβ* mRNA expression in response to infection with human cytomegalovirus^22^. Thus, the role of IFI16 during DNA sensing has remained controversial.

Here, we generated human immortalized keratinocytes lacking IFI16, in order to unambiguously determine to what extent IFI16 is required for the innate immune response to DNA in these cells. We show that IFI16 is specifically required for the innate immune response to transfected DNA and to infection with nuclear and cytosolic DNA viruses, but is dispensable for the response to poly(I:C), *in vitro* transcribed RNA, and during infection with Sendai virus. Indeed, the RNA-induced responses are frequently enhanced in the absence of IFI16, possibly due to the competition between DNA and RNA sensing pathways for downstream signalling factors such as TBK1 and IRF3. By analysing the events that follow the detection of foreign DNA in more detail, we find that IFI16 synergizes with cGAMP in the activation of STING. Our data suggest that the activation of STING relies on two independent signals from cGAMP and IFI16, and both are required for optimal STING phosphorylation and translocation, and the full activation of the resulting signalling cascades.

It is clear that in HEK293T cells, which lack many of the key components of the DNA sensing pathway, the activation of STING can be driven by cGAS and cGAMP alone (Fig. 4g), or alternatively by IFI16 in the absence of cGAS (Fig. 7e and refs 11, 13). In keratinocytes, which naturally respond to DNA, this is not the case, as both IFI16 and cGAS are required for the full activation of STING after DNA stimulation. Thus, the activation of STING is likely more complex and more tightly controlled under physiological conditions, where STING protein levels may be limiting, and additional regulatory mechanisms are likely to exist. Thus, while HEK293T cells provide a convenient model to test some of the signalling mechanisms at play, the more complex regulation of this signalling pathway will require detailed analysis in more appropriate cell systems that have evolved to respond to exogenous DNA with a high level of selectivity to prevent potentially damaging responses.

In recent years, a multitude of regulatory mechanisms that can influence STING function have been described. In addition to the conformation change caused by cGAMP binding, STING is regulated by a variety of post-translational modifications, including phosphorylation by TBK1 and other kinases^7^,^36^, ubiquitylation with K48-, K63- and K27-linked ubiquitin chains^43^,^44^,^45^,^46^,^47^, and palmitoylation^48^. These and other signals may be involved in the translocation of STING from the ER to the signalling compartments where TBK1 recruitment takes place, and further trafficking for the subsequent degradation of STING^6^,^34^. In addition, a number of positive and negative regulators that interact with STING have been described^49^,^50^,^51^, but their precise molecular function during DNA-mediated activation of STING has not yet been fully elucidated. Our data indicate that IFI16 is required for STING phosphorylation, and for STING translocation away from the ER following DNA stimulation. It would be of great interest to determine whether this effect is a direct consequence of IFI16 association, or whether the function of IFI16 is mediated by the addition or removal of a post-translational modification or the dissociation of a negative regulator. The detailed analysis of STING modifications and interaction partners following stimulation with DNA in cells lacking IFI16 or cGAS is required to provide additional insights into the precise molecular mechanisms of STING activation that is elicited by the cooperation of DNA sensors and co-factors. In this context, it would also be important to characterize the degradation of STING that usually follows its activation. Our data suggest that the absence of IFI16 causes an un-coupling of STING activation and degradation, as degradation appears to proceed normally, despite reduced levels of STING phosphorylation and trafficking in *IFI16*-deficient cells (see Supplementary Fig. 3c and Figs 6c and 7b).

It is interesting to note that a parallel study on IFI16 function in human THP-1 monocytes and primary monocyte-derived macrophages found an analogous function of IFI16 in promoting the phosphorylation of STING in response to exogenous DNA and cGAMP^52^, with a similar impairment in IFN induction in the absence of IFI16. However, the authors also show, that in this cellular context, IFI16 can perform an additional function during DNA sensing in also promoting the production of cGAMP by cGAS. Thus, there may be cell-type-specific differences in the regulation of the DNA sensing pathway.

We find that in human keratinocytes, cGAS and IFI16 function more independently of each other, only co-operating at the level of STING activation. Furthermore, while in other cell types cGAS promotes IFI16 protein expression after DNA stimulation^15^,^20^, this is not the case in human keratinocytes, where IFI16 protein levels remain unchanged over a 12 h time course after DNA transfection (see Fig. 3g).

Thus, it is conceivable that the range of IFI16 functions may depend on its relative abundance in the cell, which is particularly dynamic in monocytes and macrophages. In monocytes and THP1 cells, IFI16 protein expression is induced very strongly by differentiation, and this correlates with an increased sensitivity to exogenous DNA in those cells^10^. In these cells, IFI16 levels increase even further upon DNA stimulation, providing a positive feedback loop. This positive feedback loop is absent in human keratinocytes, which may serve to prevent excessive immune activation after localized infection. Differences in the relative expression levels of cGAS, IFI16 or other AIM2-like receptors may also account for some of the observed differences between mouse and human cells, and between different mouse strains^22^. While we and others provide strong evidence for an involvement of IFI16 in DNA sensing in human cells, the function of IFI16 homologues in mice may need to be investigated further.

In summary, we show here that cGAS and IFI16 cooperate in the sensing of intracellular DNA in human keratinocytes. While we still observe a weak transcriptional response to exogenous DNA in the absence of IFI16 in these cells, IFI16 is critical for the full activation of STING, and cooperates with cGAMP in the activation of this key signalling adaptor. The integration of IFI16 into the cGAS-cGAMP-STING signalling cascade provides a further level of regulation of STING activation that may be important to prevent the spurious activation of the innate immune system.

## Methods

### Cells and viruses

Immortalized human keratinocytes (HaCaT), MRC-5 human embryonic lung fibroblasts and immortalized human embryonic kidney HEK293T cells were cultured in Dulbecco's Modified Eagle's Medium (DMEM, Gibco) supplemented with 10% (v/v) FCS and 50 μg ml^−1^ gentamicin. Primary human keratinocytes from adult donors were obtained from Lonza, and grown in KGM-Gold Keratinocyte Basal Medium supplemented with KGM-Gold SingleQuots (Lonza). Cell lines were regularly tested for mycoplasma contamination.

Sendai virus (SeV, strain Cantell) was kindly provided by R. Randall (University of St. Andrews, UK). Vaccinia virus with RFP-tagged A3L protein (VACV-RFP)^53^ was propagated in RK13 cells and sucrose-purified. MVA was kindly provided by B. Ferguson (University of Cambridge, UK). MVA was propagated in BHK cells and sucrose-purified. GFP-tagged Herpes Simplex Virus 1 (HSV-1-GFP) was kindly provided by F. Grey (The Roslin Institute, University of Edinburgh, UK) and propagated in Vero cells. All viruses were titrated on BSC cells.

### Generation of *IFI16*−/− and *cGAS* −/− HaCaT cells

HaCaT cells lacking cGAS or IFI16 were generated using CRISPR-Cas9 nickase or TALE nuclease technology, respectively. Plasmids encoding left and right TALEN arms, or Cas9 nickase and two guide RNAs, were transfected into HaCaT cells using electroporation with the Neon system (Life Technologies). Cells were selected for 48 h with puromycin, then allowed to recover and seeded as single cells in 96-well plates. DNA was extracted from individual colonies using Quickextract DNA extraction solution (EpiBio), and screened for modifications of the target site, using high resolution melting analysis on a LifeCycler 96 system (Roche), using LightCycler480 High Resolution Melting master mix (Roche). Candidate clones displaying mutated target sites were screened for lack of protein expression by western blotting of IFI16 or cGAS and β-actin, and by immunofluorescence analysis to confirm homogeneity of cell clones.

### Virus infection

HaCaT cells were seeded 24 h before infection and were infected with VACV or MVA in DMEM supplemented with 2.5% (v/v) FCS for 1 h, before replacing the inoculum with DMEM containing 2.5% (v/v) FCS. HSV-1-GFP infections (MOI=1) were performed in serum-free DMEM for 1 h, followed by the maintenance of cells in complete DMEM containing 10% (v/v) FCS. Infections with a SeV preparation containing defective viral particles was carried out in serum-free DMEM for 1 h, followed by replacement of the medium with complete DMEM containing 10% (v/v) FCS. Infections were allowed to proceed for 6 h, unless indicated otherwise.

### Transfection of nucleic acids and cGAMP

Cells were seeded at 1–1.5 × 10^5^ cells per ml 24 h before transfection, and stimulated with 1 μg ml^−1^ HT DNA (HT DNA, Sigma), a double-stranded 70mer oligonucleotide derived from VACV (5′-CCATCAGAAAGAGGTTTAATATTTTTGTGAGACCATCGAAGAGAGAAAGAGATAAAACTTTTTTACGACT-3′)^10^, Y-G3 DNA (5′-GGGGAACTCCAGCAGGACCATTGGGG-3′) or Y-C3 DNA (5′-CCCGAACTCCAGCAGGACCATTGCCC-3′)^24^. DNA oligonucleotides were synthesized by Biofins Genomics, Germany. *In vitro* transcribed RNA containing a 5′-triphosphate was generated using the MEGAScript T7 transcription kit (Thermo Fisher) with pcDNA3.1: EGFP as template. 50 ng ml^−1^ of *in vitro* transcribed RNA and 100 ng ml^−1^ poly(I:C) (Sigma) were used, unless indicated otherwise. 2′3′ cGAMP (Invivogen) or cyclic di-AMP (Invivogen) were transfected at 20 and 100 μg ml^−1^, respectively. All transfections were carried out with 1 μl Lipofectamine 2000 (Life Technologies) per ml medium.

Transfection by digitonin permabilization was carried out in a buffer containing 50 mM HEPES (pH 7), 100 mM KCl, 3 mM MgCl_2_, 0.1 mM DTT, 85 mM saccharose, 1 mM ATP, 0.1 mM GTP and 0.2% (v/v) BSA. 25 μg ml^−1^ HT DNA (Sigma). 15 μM 2′3′ cGAMP or 2′3′ cGAM(PS)_2_ (both Invivogen) was transfected using 5 μg ml^−1^ digitonin in permeabilization buffer for 10 min at 37 °C before replacing the permabilization buffer with DMEM containing 10% (v/v) FCS.

### Quantitative real-time PCR (qRT-PCR)

RNA was extracted using HighPure RNA Isolation Kits (Roche), and cDNA was synthesized using the iScript cDNA Synthesis Kit (Bio-Rad Laboratories). Real-time PCR amplification was performed in a 10 μl reaction containing FastStart Universal SYBR Green Master Mix (Roche) on a LifeCycler 96 system (Roche). The real-time PCR program was as follows: initial denaturation at 95 °C for 600 s; 40 cycles of 95 °C for 10 s then 60 °C for 30 s; followed by a melt curve step. Quantification cycle (*C*_q_) of mRNAs of interest were normalized to *C*_q_ of β-actin reference mRNA and data was expressed as fold change over mock treatment. Primers were synthesized by Eurofins Genomics. Primer sequences were: *β-actin* forward (FWD): 5′-CGCGAGAGAAGATGACCCAG;ATC-3′; *β-actin* reverse (REV): 5′-GCCAGAGGCGTACAGGGATA-3′; *IFNβ* FWD: 5′-ACGCCGCATTGACCATCTAT-3′; *IFNβ* REV: 5′-GTCTCATTCCAGCCAGTGCT-3′; *CXCL10* FWD: 5′-AGCAGAGGAACCTCCAGTCT-3′; *CXCL10* REV: 5′-AGGTACTCCTTGAATGCCACT-3′; *CCL5* FWD: 5′-CTGCTTTGCCTACATTGCCC-3′; *CCL5* REV: 5′-TCGGGTGACAAAGACGACTG-3′; *ISG56* FWD: 5′-CAAAGGGCAAAACGAGGCAG-3′; *ISG56* REV: 5′-CCCAGGCATAGTTTCCCCAG-3′; *IL6* FWD: 5′-CAGCCCTGAGAAAGGAGACAT-3′, *IL6* REV: 5′-GGTTCAGGTTGTTTTCTGCCA-3′.

### ELISA

For the quantification of secreted chemokines by ELISA, cells were stimulated for 24 h as indicated. Supernatants were harvested and secreted CCL5 or CXCL10 protein levels were quantified using Human CCL5/Rantes (DY278) and Human CXCL10/IP-10 (DY266) DuoSet ELISA kits (R&D Systems) according to manufacturer's instructions. Absorbance was measured at 450 nm and corrected against absorbance at 570 nm.

### Western blotting and antibodies

For western blotting, cells were harvested in lysis buffer containing 50 mM Tris (pH 7.4), 150 mM NaCl, 30 mM NaF, 5 mM EDTA, 10% (v/v) glycerol, 40 mM β-glycerophosphate, 1% (v/v) Triton X-100, 1 mM sodium orthovanodate, 0.1 mM phenylmethanesulfonylfluoride and 0.07 μM aprotinin. Proteins were separated by SDS–PAGE and transferred to polyvinylidene (PVDF) membranes using semi-dry transfer. Membranes were blocked with 5% (w/v) non-fat milk in PBS containing 0.1% (v/v) Tween-20 (PBS-T) for 1 h before incubation with antibodies. Western blots using antibodies against phosphorylated proteins were performed with TBS containing 0.1% (v/v) Tween-20 and 5% bovine serum albumin (BSA).

The antibodies used were anti-IFI16 (1G7, Santa Cruz Biotechnology), anti-cGAS (HPA031700, Sigma Aldrich), anti-STING (D2P2F, Cell Signaling Technology), anti-TBK1 (D1B4, Cell Signaling Technology), anti-IRF3 (D6I4C, Cell Signaling Technology), anti-β-actin (A2228, Sigma Aldrich), anti-phospho(Ser172)-TBK1 (D52C2, Cell Signaling Technology) and anti-phospho(Ser396)-IRF3 (4D4G, Cell Signaling Technology). Primary antibodies were used at a dilution of 1:1,000. Secondary horse radish peroxidase-coupled anti-mouse (7076 S) and anti-rabbit (7074 S) antibodies were from Cell Signaling Technology and used at a dilution of 1:3,000. Full immunoblots including size markers are shown in Supplementary Fig. 6.

### Luciferase assays

HEK293T cells were seeded in 96-well plates at 1 × 10^5^ cells per ml, and transfected with 60 ng of a firefly luciferase construct under the control of an *IFNβ* promoter (IFNβ-luciferase, obtained from T Taniguchi, University of Tokyo) and 60 ng pGL3-Renilla luciferase transfection control^10^ per well. In addition, pcDNA3.1:STING-FLAG (kindly provided by Lei Jin, Albany Medical Centre) and cGAS or IFI16 expression constructs were co-transfected as indicated. Empty vector (pCMV-HA, Clontec) was added to keep amounts of DNA constant. Transfections were carried out using 0.8 μl GeneJuice Transfection Reagent (Merck Millipore) per well, and cells were lysed in Passive Lysis Buffer (Merck Millipore) 24 h post transfection. Firefly luciferase activity was measured and normalized to Renilla luciferase activity in each sample. IFI16 truncations (Pyrin domain, aa 1–91; HINb domain, aa 507–730) were cloned into pIRESpuro2 containing an N-terminal HA tag.

### Co-culture of HEK293T and HaCaT cells

For the co-culture with cGAS-expressing cells, HEK293T cells were transfected with pCMV6-Entry:cGAS-myc-FLAG (OriGene) or empty vector for 6 h using GeneJuice Transfection Reagent (Merck Millipore). cGAS-expressing HEK293T and wild-type or *IFI16* −/− HaCaT cells were seeded together in 12-well plates at a ratio of 1:4 (HEK293T:HaCaT) at a total of 1.5 × 10^5^ cells per ml. RNA and protein samples were harvested after 18 h of co-culture.

### siRNA transfection

Pools of four dual strand modified siRNAs were obtained from GE Dharmacon (ON-TARGETplus SMARTpool siRNA), and used as pool or individually, as indicated. Primary human keratinocytes or MRC-5 fibroblasts were seeded in 24-well plates at 1.5 × 10^5^ cells ml^−1^ and transfected with 5 nM of non-targeting siRNA pools or IFI16-targeting siRNA using 3 μl ml^−1^ of Lipofectamine RNAimax (Life Technologies). Cells were stimulated 48 h after treatment with siRNA.

### Immunofluorescence and confocal microscopy

Cells were seeded on coverslips 24 h before stimulation with DNA or infection as indicated. Cells were washed with PBS, and fixed in methanol at −20 °C. Cells were permeabilized for 12 min in 0.5% Triton-X in PBS, washed in PBS, and incubated for 1 h in blocking solution (5% FBS, 0.2% Tween-20 in PBS). Cells were stained with primary antibodies (1:600 in blocking solution) at room temperature over night. Primary antibodies used were anti-IFI16 (1G7, Santa Cruz Biotechnology), anti-STING (D2P2F, Cell Signaling Technology) and anti-IRF3 (D6I4C, Cell Signaling Technology). Coverslips were washed in PBS, and incubated for 3 h with fluorescently labelled secondary antibodies, used at a dilution of 1:1,500 in blocking solution. Anti-mouse IgG labelled with AlexaFluor647 (A21236) or AlexaFluor488 (A11029), and anti-rabbit IgG labelled with AlexaFluor488 (A11034) were from Life Technologies. Coverslips were washed in PBS and mounted in MOWIOL 4–88 containing 1 μg ml^−1^ DAPI. Images were obtained using a × 100 oil immersion objective on a LSM710 laser scanning microscope (Zeiss).

### mRNA reconstitution

DNA plasmids pcDNA3.1(+):GFP or pcDNA3.1(+):IFI16 were used as templates for the *in vitro* synthesis of capped and polyadenylated mRNA using the mMESSAGE mMACHINE T7 Transcription Kit (ThermoScientific). *IFI16* −/− HaCaT cells were seeded at 1 × 10^5^ cells/ml on coverslips 24 h before transfection with 1 μg ml^−1^ *GFP* mRNA or *IFI16* mRNA for 6 h using using 1 μl ml^−1^ of Lipofectamine 2000 (Life Technologies). Cells were then stimulated with 5 μg ml^−1^ HT DNA (Sigma) for 1 h.

### cGAMP detection by LC-MS

5 × 10^6^ HaCaT cells per sample were lysed in cold 80% methanol, followed by the addition of 0.45 pmol cyclic-di-AMP, as internal spike-in to control for losses in sample preparation and injection. Cell debris was removed by centrifugation, samples were dried by vacuum centrifugation, and then subjected to three rounds of butanol:water extraction. Dried samples were resuspended in 1 ml H_2_O and subjected to solid phase extraction using HyperSep Aminopropyl columns (ThermoFisher). Columns were activated using 80% methanol before the addition of samples. The columns were then washed twice with a solution of 2% (v/v) acetic acid/80% (v/v) methanol. Elution was performed using a solution of 4% (v/v) ammonium hydroxide/80% (v/v) methanol. Samples were dried again by vacuum centrifugation and resuspended in 40 μl H_2_O for analysis by liquid chromatography and mass spectrometry (LC-MS).

cGAMP levels were measured using a TSQ Quantiva interfaced with Ultimate 3000 Liquid Chromatography system (ThermoScientific), equipped with a porous graphitic carbon column (HyperCarb 30 × 1 mm ID 3 μm; Part No: C-35003-031030, Thermo-Scientific). Mobile phase buffer A consisted of 0.3% (v/v) formic acid adjusted to pH 9 with ammonia before a 1/10 dilution. Mobile phase buffer B was 80% (v/v) acetonitrile. The column was maintained at a controlled temperature of 30 °C and was equilibrated with 13% buffer B for 15 min at a constant flow rate of 0.06 ml min^−1^. Aliquots of 13 μl of each sample were loaded onto the column and compounds were eluted from the column with a linear gradient of 13–80% buffer B over 20 min. Buffer B was then increased to 100% for 5 min and the column was washed for a further 5 min with Buffer B. Eluents were sprayed into the TSQ Quantiva using Ion Max NG ion source with ion transfer tube temperature set to 350 °C and vaporizer temperature 125 °C. The TSQ Quantiva was run in negative mode with a spray voltage of 2,600 V, sheath gas 40 and Aux gas 10. cGAMP and spiked in cyclic di-AMP levels were measured using multiple reaction monitoring mode with optimized collision energies and radio frequencies previously determined by infusing pure compounds. Three transitions (673.05>328.03, 673.05>343.92 and 673.06>522.00) were used to monitor cGAMP and one transition (657.07>328.03) was used to detect cyclic di-AMP.

### Co-immunoprecipitation

Cells were lysed in IP lysis buffer (25 mM Tris-HCl pH 7.4, 150 mM NaCl, 1% NP-40, 1 mM EDTA, 50 mM NaF and 5% glycerol), supplemented with Complete protease inhibitor cocktail (Roche). Samples were pre-cleared by centrifugation at 2,000 *g* for 10 min before incubation with antibodies overnight at 4 °C, followed by the addition of protein G beads (ThermoFisher) for 3 h. Immunoprecipitates were washed three times with the IP lysis buffer. Bound proteins were eluted by boiling in SDS-sample buffer and analysed by western blot.

### Treatment with phosphatase and benzonase

For phosphatase treatment, immunoprecipitates containing STING were incubated with 25 U λ phosphatase for 1 h at 30 °C. For treatment with benzonase, immunoprecipitates were washed in lysis buffer without EDTA, and incubated in 100 μl benzonase reaction buffer (50 mM Tris-Cl, pH 8, 2 mM MgCl2, 150 mM NaCl) with 1.5 U μl^−1^ benzonase for 1 h at 37 °C. Immunoprecipitates were washed twice in lysis buffer and analysed by SDS–PAGE and western blotting.

### Statistical analysis

Results from real-time PCR analysis, luciferase assays, ELISA and cGAMP quantification are presented as averages of triplicate samples with error bars representing s.d. Data were subjected to a multiple *t*-test statistical analysis with the Holm-Sidak method. *P<0.05, **P<0.01, ***P<0.001.

### Data availability

The data that support the findings of this study are available from the corresponding author upon request.

## Additional information

**How to cite this article:** Almine, J. F. *et al*. IFI16 and cGAS cooperate in the activation of STING during DNA sensing in human keratinocytes. *Nat. Commun.* **8**, 14392 doi: 10.1038/ncomms14392 (2017).

**Publisher's note:** Springer Nature remains neutral with regard to jurisdictional claims in published maps and institutional affiliations.

## Supplementary Material

Supplementary Information Supplementary Figures
