# Supplementary material for: IFI16 and cGAS cooperate in the activation of STING during DNA sensing in human keratinocytes
Source: Nat Commun. 2017 Feb 13;8:14392. doi: 10.1038/ncomms14392 (PMC5316833; doi:10.1038/ncomms14392)

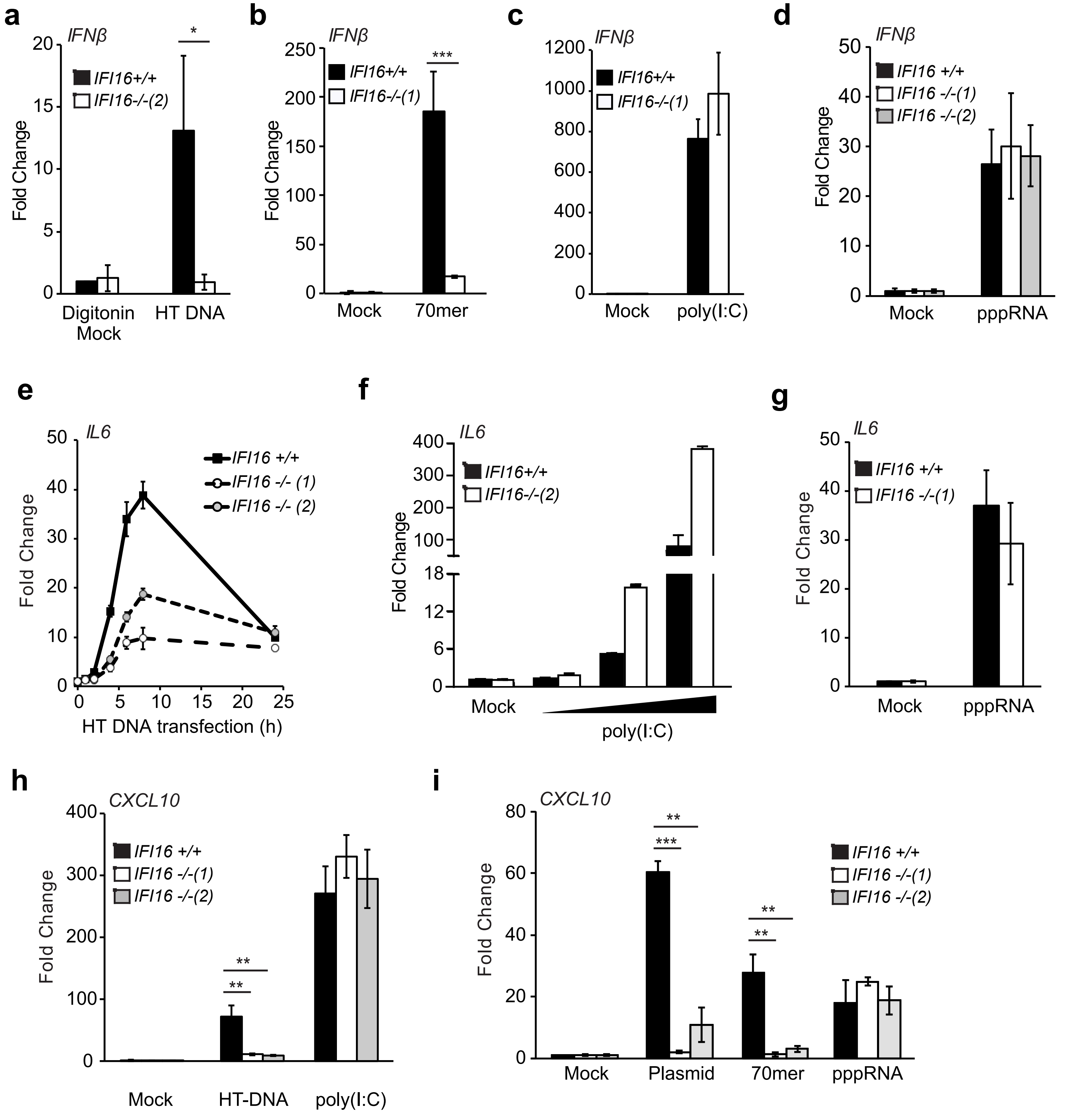

Supplementary Figure 1. IFI16 is required for DNA, but not RNA sensing in HaCaT keratinocytes.
(a-i) qRT-PCR analysis of mRNA expression levels normalised to *β-actin* mRNA and mock treatment in wild type (*IFI16* +/+) HaCaT keratinocytes or two IFI16-deficient HaCaT clones, *IFI16* -/- (1) or (2), as indicated. Data are presented as mean values of biological triplicates. Error bars indicate standard deviation (sd). * $p<0.05$, ** $p<0.01$, *** $p<0.001$ Student's t-test. **(a)** Cells were permeabilised with digitonin, and infused with 25 μg/ml herring testis (HT) DNA for 6 h. *IFN-β* mRNA expression levels were quantified by qRT-PCR. **(b)** *IFN-β* mRNA expression 6 h post mock transfection, or transfection with 1 μg/ml 70mer oligonucleotide. **(c)** I*FN-β* mRNA expression after transfection of 100 ng/ml poly(I:C). **(d) IFN-β** mRNA levels after transfection with 50ng/ml *in vitro* transcribed *GFP* mRNA containing 5' triphosphate groups (pppRNA). **(e)** Time course analysis of *IL-6* mRNA expression following transfection with 1 μg/ml HT DNA for the times indicated. **(f)** *IL-6* mRNA expression 6 h post transfection with 1, 10 or 100 ng/ml poly(I:C). **(g)** *IL-6* mRNA expression 6 h post transfection with 50 ng/ml *in vitro* transcribed mRNA (pppRNA). **(h)** *CXCL10* mRNA 6 h post transfection with 1 μg/ml HT DNA or 100 ng/ml poly(I:C). **(i)** *CXCL10* mRNA expression 6 h post transfection with 1 μg/ml plasmid DNA or 70mer oligoncleotide, or 50 ng/ml *in vitro* transcribed RNA (pppRNA).

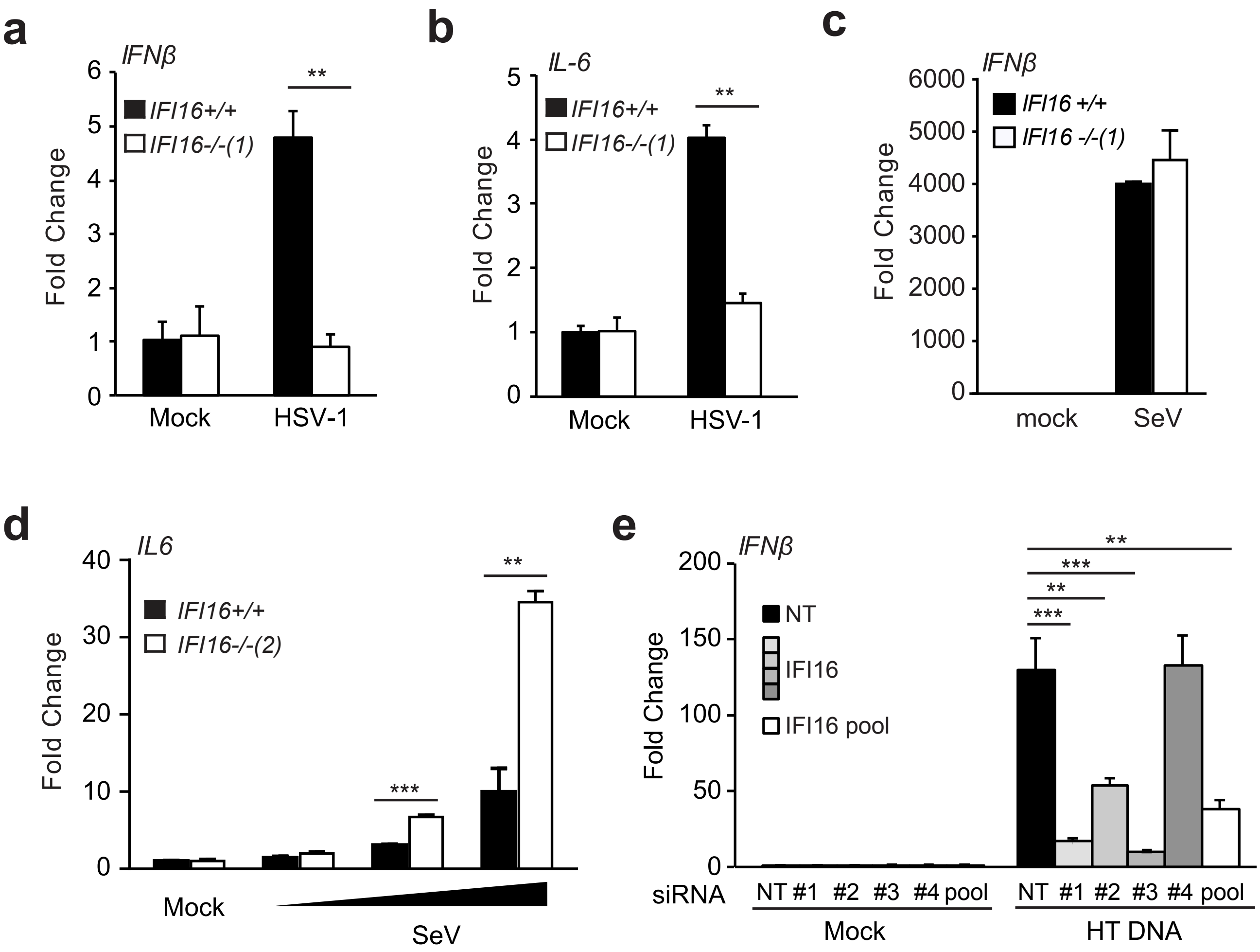

Supplementary Figure 2. IFI16 is required for the response to DNA viruses
(a,b) Wild type (IFI16 +/+) or IFI16-deficient HaCaT cells were infected with HSV-1 (MOI=1) for 6 h. IFN-β (a) or IL-6 (b) mRNA expression was analysed by qRT-PCR. **(c)** HaCaT cells were infected with a preparation of Sendai virus containing defective viral particles for 6h, and IFN-β mRNA expression was measured by qRT-PCR. **(d)** HaCaT cells were infected with a preparation of Sendai virus, at dilutions of 1:20,000, 1:2,000 and 1:200. After 6h, levels of IL-6 mRNA were quantified by qRT-PCR. **(e)** MRC-5 human embryonic lung fibroblasts were treated with a non-targeting (NT) or IFI16-targeting pool of siRNAs, or the four IFI16-targeting siRNAs individually as indicated. Expression of IFN-β mRNA was quantified following transfection of 1 μg/ml HT DNA for 6h. All data are presented as mean values of biological triplicates. Error bars indicate standard deviation (sd). * p<0.05, ** p<0.01, *** p<0.001 Student's t-test.

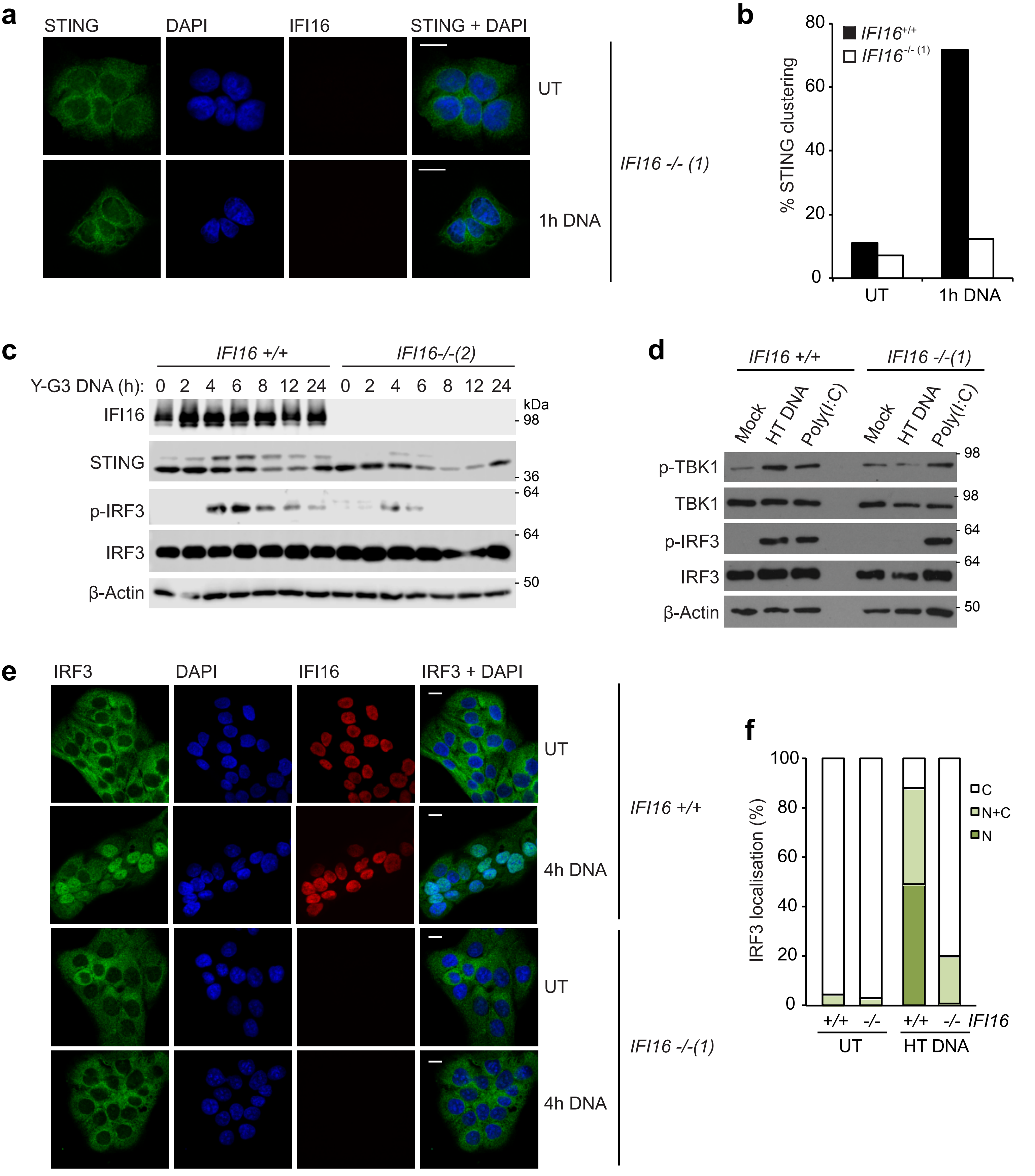

Supplementary Figure 3. IFI16 acts 'upstream' of STING, TBK1 and IRF3.
(a) Wild type or IFI16-deficient HaCaT cells were treated with 1 µg/ml HT DNA, and STING clustering was observed by confocal microscopy. Cells were stained for STING (green) and IFI16 (red). **(b)** Cells as in (a) were scored for STING clustering. At least 200 cells were counted per sample. **(c)** *IFI16* +/+ or -/- HaCaT cells were treated with Y-G3 DNA oligonucleotide for the times indicated. STING and IRF3 phosphorylation was assessed by Western blotting. **(d)** Cells were treated with 1 µg/ml HT DNA or 100 ng/ml poly(I:C) for 4h. TBK1 and IRF3 phosphorylation was assessed by Western blotting. **(e)** Wild type or IFI16-deficient HaCaT cells were stimulated with 1 µg/ml HT DNA for 4h. IRF3 translocation to the nucleus was observed by confocal microscopy. Cells were stained for IRF3 (green) and IFI16 (red), nuclear DNA is stained with DAPI (blue). **(f)** Cells as in (e) were scored for predominantly cytosolic (C), predominantly nuclear (N) or even nuclear and cytosolic distribution of IRF3. At least 200 cells were counted per sample. Scale bars: 20 µm.

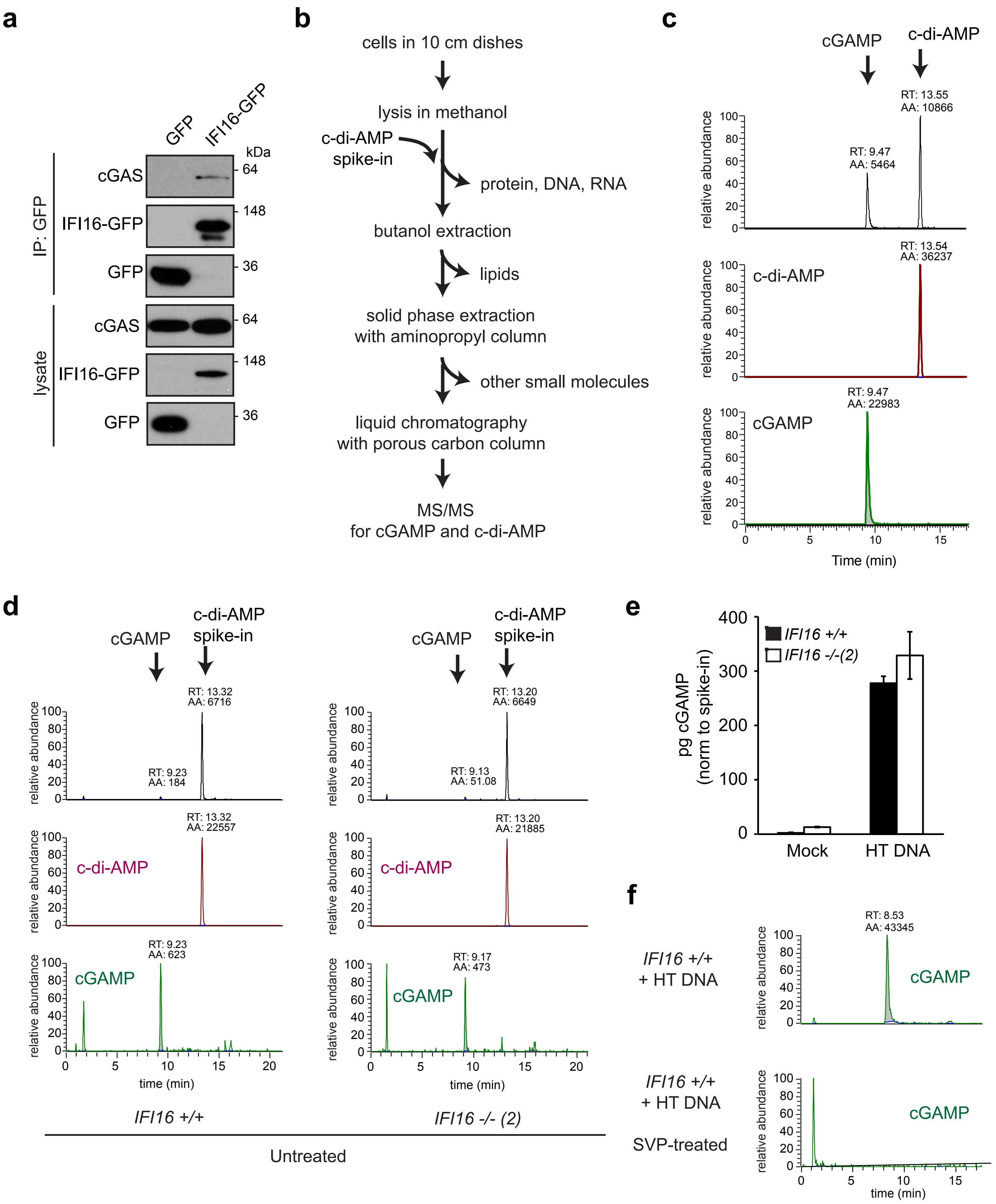

Supplementary Figure 4. IFI16 does not affect cGAMP production.
(a) HEK293 Trex FlpIn cells were induced to express IFI16-GFP or GFP alone by treatment with 1 µg/ml tetracyclin for 18 h, prior to immunoprecipitation with anti-GFP antibody. cGAS and GFP fusion proteins were detected by immunoblotting. **(b)** Schematic representation of sample preparation for analysis by LC-MS. **(c)** Total and extracted ion chromatogram of 50 pg synthetic cGAMP and cyclic di-AMP standards. **(d)** Quantification of cGAMP by LC-MS in untreated wild type (*IFI16* +/+) or *IFI16* -/- HaCaT cells. **(e)** Production of endogenpus cGAMP quantified by LC-MS 2 h post transfection of 1 µg/ml HT DNA. Data are presented as mean values of triplicate samples; error bars represent standard deviations. **(f)** cGAMP extracted ion chromatogram of wild type HaCaT cells stimulated with 1 µg/ml HT DNA for 4h (top) and from parallel lysates treated with 0.05 U snake venom phosphodiesterase

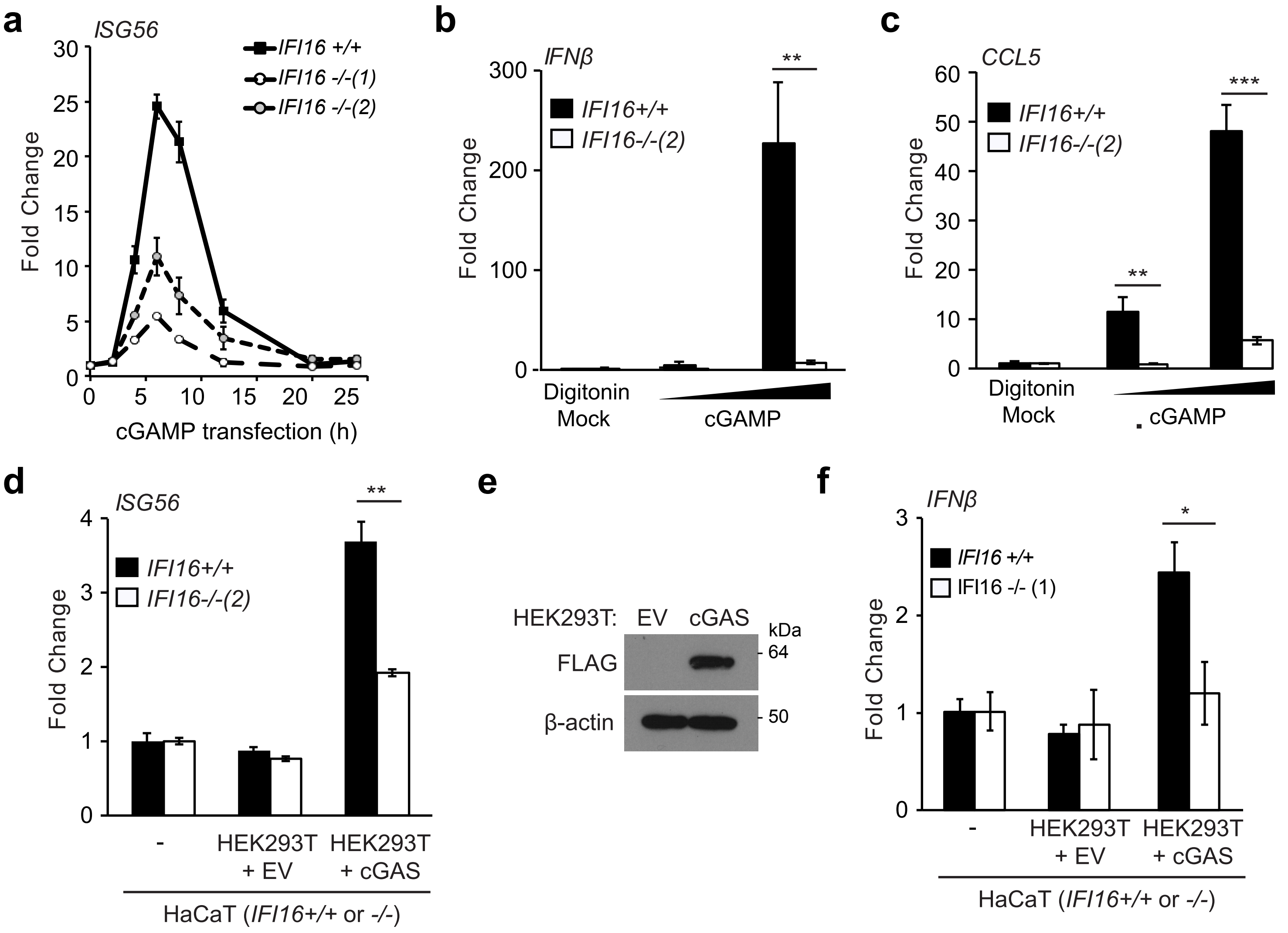

Supplementary Figure 5. IFI16 is required for the response to cGAMP.
(a) *IFI16* +/+ and two clones of *IFI16* -/- HaCaT cells were transfected with 20 µg/ml synthetic cGAMP, and *ISG56* mRNA induction was analysed by qRT-PCR at the time points indicated. **(b, c)** *IFI16* +/+ and *IFI16* -/- HaCaT cells were permeabilised with 5 µg/ml digitonin in the presence of 15 µM 2'3' cGAMP. *IFNβ* **(b)** and *CCL5* **(c)** mRNA levels were analysed after 6 h and normalised to *β-actin* mRNA levels and mock permeabilisation. **(d)** qRT-PCR analysis of *ISG56* mRNA induction in HaCaT cells grown in monoculture (-) or co-cultured with HEK293T cells transfected with a cGAS expression construct or empty vector (EV) as indicated. **(e)** *IFI16* +/+ or *IFI16* -/- (1) HaCaT cells were co-cultured with HEK293T cells expressing either cGAS-FLAG or empty vector (EV). cGAS protein expression was verified by Western blot. **(f)** qRT-PCR analysis of *IFN-β* mRNA expression from *IFI16* +/+ or *IFI16* -/- HaCaT cells cultured on their own (-) or co-cultured with HEK293T cells transfected with as cGAS expression construct or empty vector (EV) as indicated. Data are presented as mean values of biological triplicates. Error bars indicate standard deviation (sd). * p<0.05, ** p<0.01, *** p<0.001 Student’s t-test.

Supplementary Figure 6. Uncropped immunoblots from Figures 1 - 7.

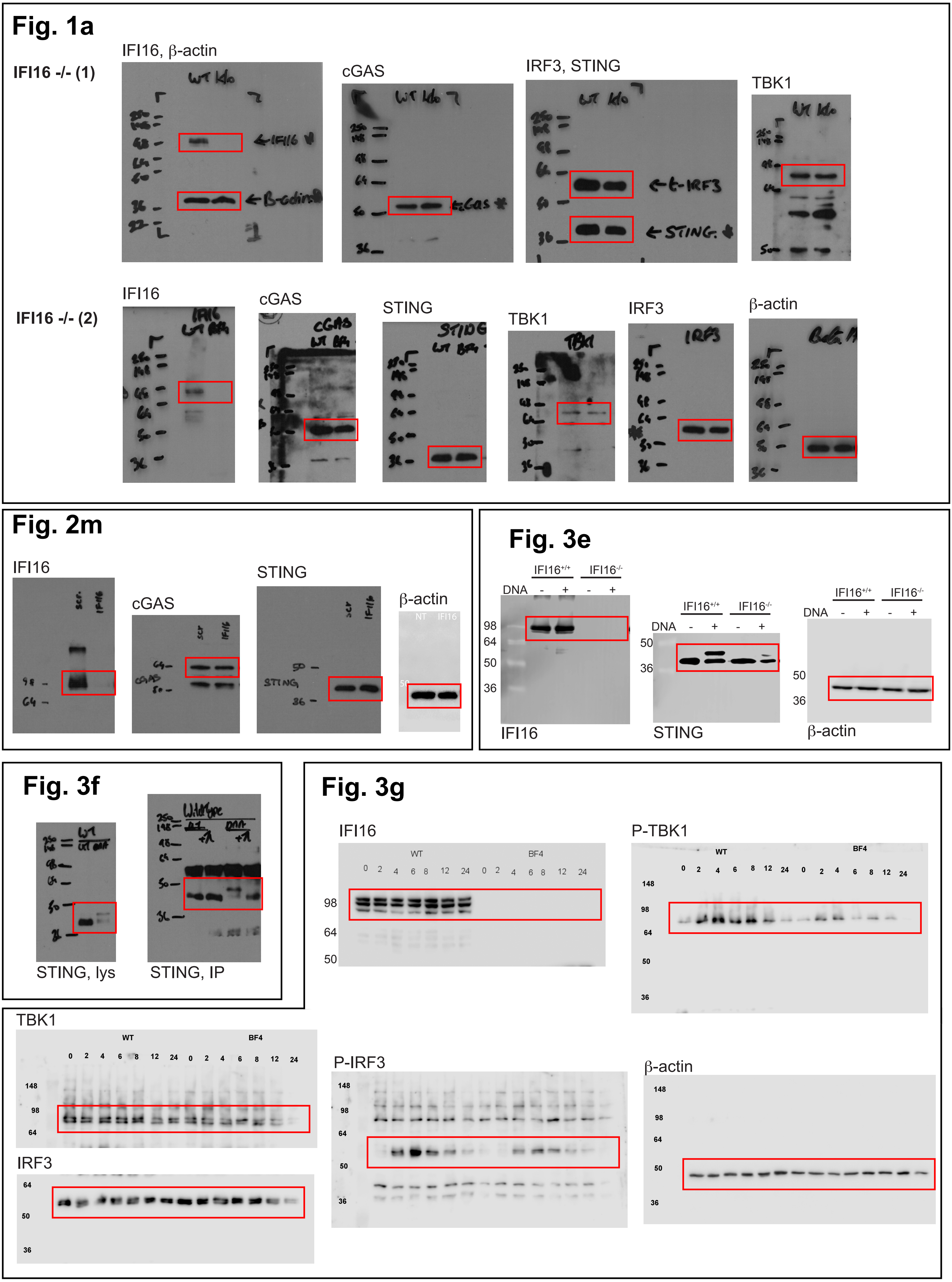

Fig. 4a

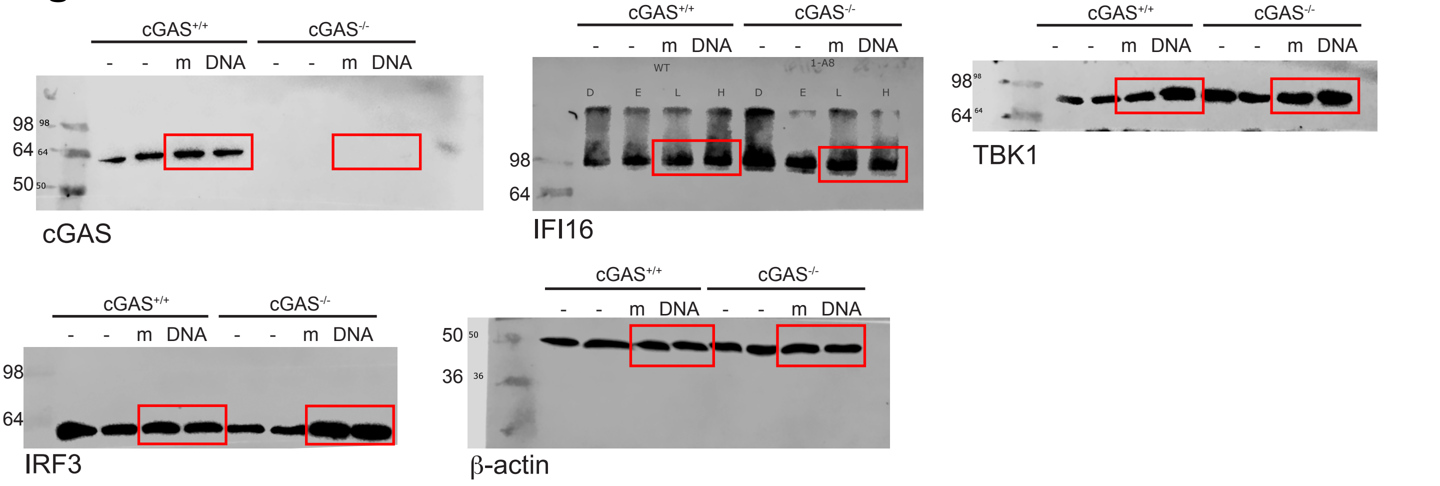

Fig. 5a

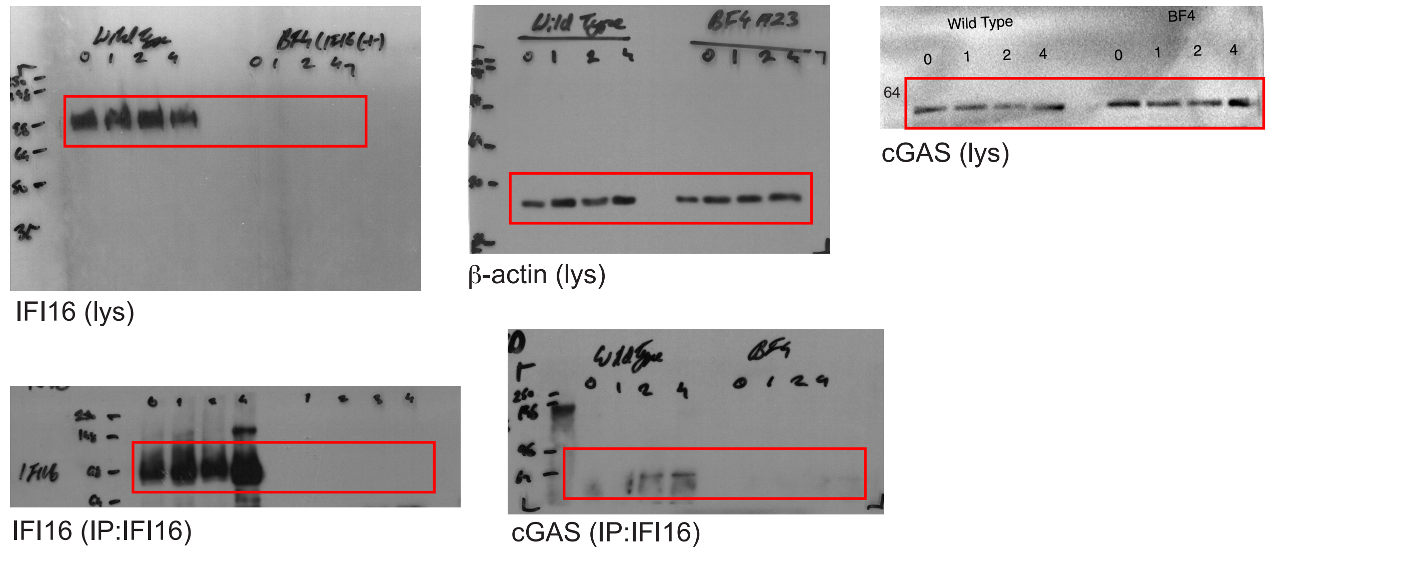

Fig. 5b

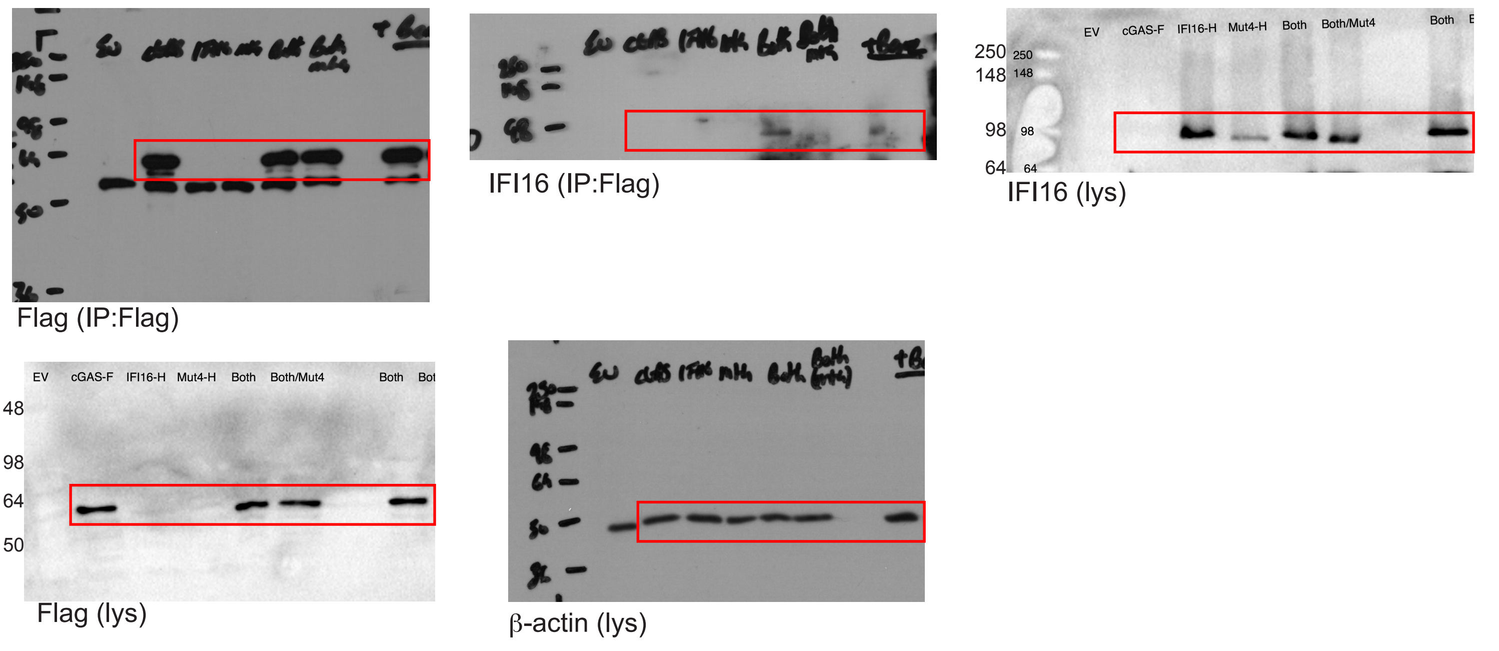

Fig. 6c

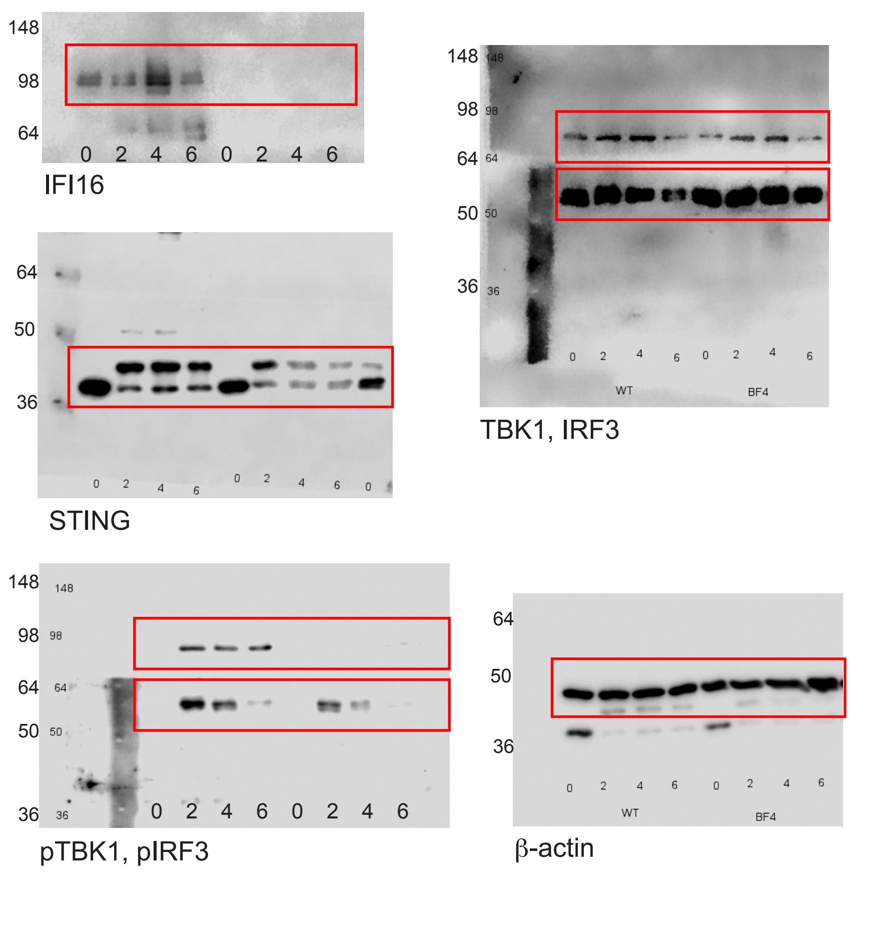

Fig. 6g

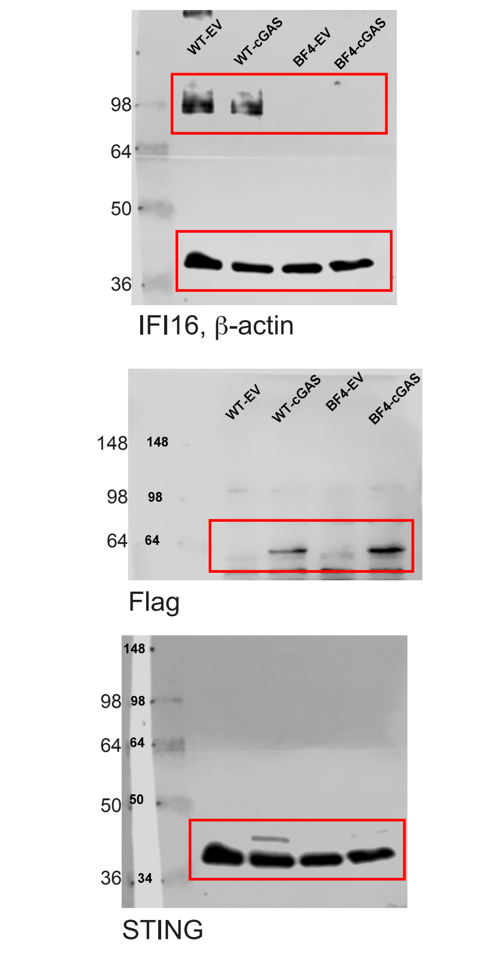

Fig. 7b

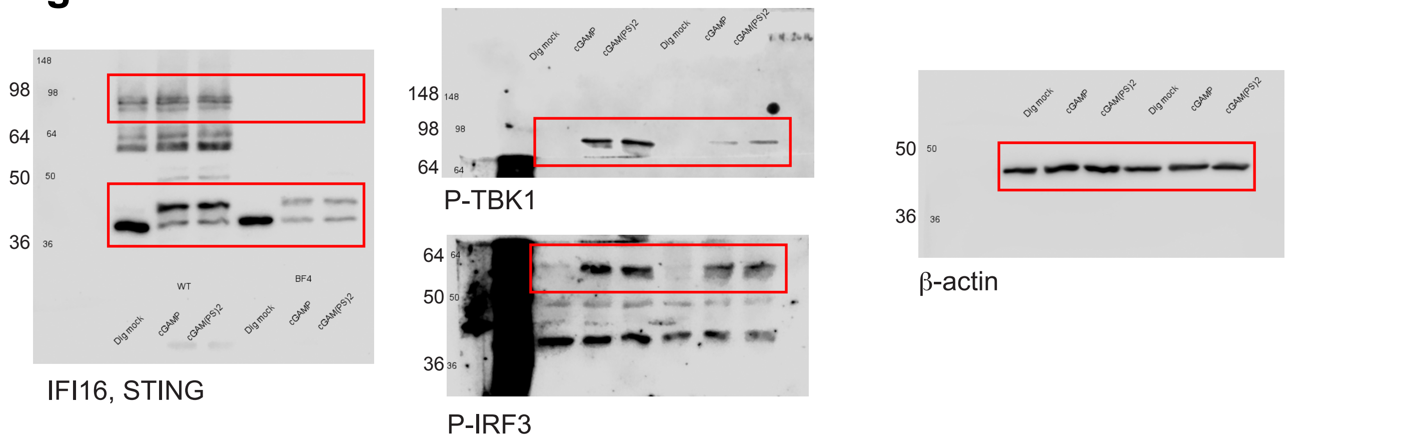

Fig. 7d

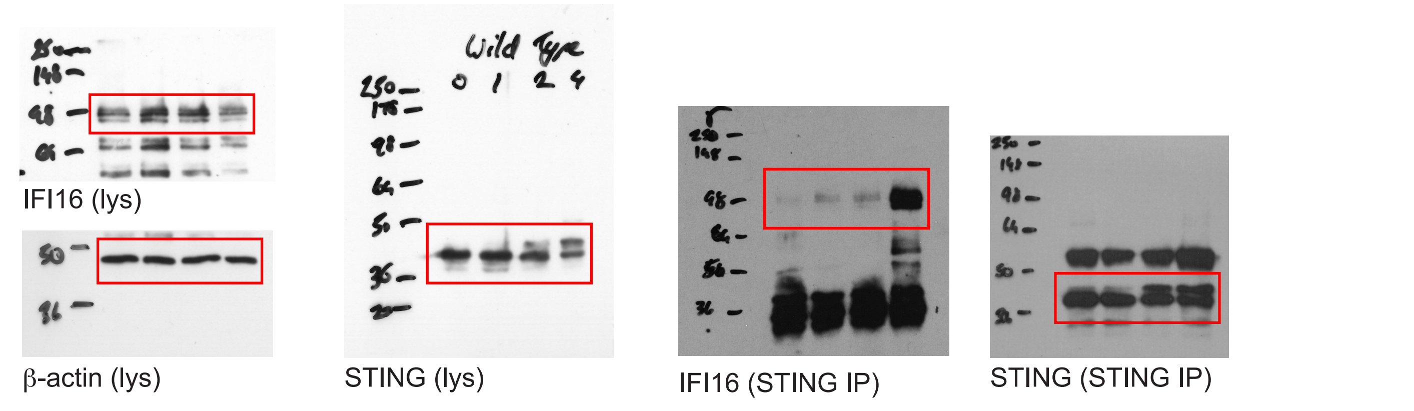

Supplement: Supplementary Information — Supplementary Figures [file ncomms14392-s1.pdf]
